# The Adenylate-Forming Enzymes AfeA and TmpB Are Involved in *Aspergillus nidulans* Self-Communication during Asexual Development

**DOI:** 10.3389/fmicb.2016.00353

**Published:** 2016-03-23

**Authors:** Gabriela Soid-Raggi, Olivia Sánchez, Jose L. Ramos-Balderas, Jesús Aguirre

**Affiliations:** Departamento de Biología Celular y del Desarrollo, Instituto de Fisiología Celular, Universidad Nacional Autónoma de MéxicoCiudad de México, Mexico

**Keywords:** coumarate ligase-like, NRPS, secondary metabolism, colony development

## Abstract

*Aspergillus nidulans* asexual sporulation (conidiation) is triggered by different environmental signals and involves the differentiation of specialized structures called conidiophores. The elimination of genes *flbA-E, fluG*, and *tmpA* results in a *fluffy* phenotype characterized by delayed conidiophore development and decreased expression of the conidiation essential gene *brlA*. While *flbA-E* encode regulatory proteins, *fluG* and *tmpA* encode enzymes involved in the biosynthesis of independent signals needed for normal conidiation. Here we identify *afeA* and *tmpB* as new genes encoding members the adenylate-forming enzyme superfamily, whose inactivation cause different *fluffy* phenotypes and decreased conidiation and *brlA* expression. AfeA is most similar to unknown function coumarate ligase-like (4CL-Lk) enzymes and consistent with this, a K544N active site modification eliminates AfeA function. TmpB, identified previously as a larger homolog of the oxidoreductase TmpA, contains a NRPS-type adenylation domain. A high degree of synteny in the *afeA-tmpA* and *tmpB* regions in the Aspergilli suggests that these genes are part of conserved gene clusters. *afeA, tmpA*, and *tmpB* double and triple mutant analysis as well as *afeA* overexpression experiments indicate that TmpA and AfeA act in the same conidiation pathway, with TmpB acting in a different pathway. Fluorescent protein tagging shows that functional versions of AfeA are localized in lipid bodies and the plasma membrane, while TmpA and TmpB are localized at the plasma membrane. We propose that AfeA participates in the biosynthesis of an acylated compound, either a *p*-cuomaryl type or a fatty acid compound, which might be oxidized by TmpA and/or TmpB, while TmpB adenylation domain would be involved in the activation of a hydrophobic amino acid, which in turn would be oxidized by the TmpB oxidoreductase domain. Both, AfeA-TmpA and TmpB signals are involved in self-communication and reproduction in *A. nidulans*.

## Introduction

Cell-cell communication is a process central to environmental sensing and development. Filamentous fungi use different extracellular chemical signals for self and non-self communication during their life cycle. Some signals function as auto-inhibitors of spore germination to prevent spore overcrowding, others coordinate growth through regulation of cell-cell fusion events and the establishment of a mycelial network, while others regulate asexual and sexual reproduction. However, only a few of such signals have been characterized in terms of their structure and/or biosynthesis (reviewed in Leeder et al., 2011; Ugalde and Rodriguez-Urra, 2014).

In filamentous fungi development and secondary metabolism are often interrelated. That is the case in *Aspergillus nidulans*, where asexual and sexual development (Butnick et al., 1984), as well as secondary metabolism (Tsitsigiannis and Keller, 2006), are regulated by oxylipins, collectively called the psi factor, derived from fatty acid oxidation (Tsitsigiannis and Keller, 2007). Likewise, the partial inactivation of the phosphopantetheinyl transferase CfwA/NpgA, required for activation of all polyketide synthases (PKSs) and non-ribosomal peptide synthetases (NRPSs), results in an almost complete lack of asexual reproduction (Márquez-Fernández et al., 2007).

*A. nidulans* asexual sporulation (conidiation) is induced by air exposure (Timberlake and Clutterbuck, 1994) or nutrient starvation (Skromne et al., 1995), and depends on the expression of the *brlA* gene (Clutterbuck, 1969; Adams et al., 1988; Aguirre, 1993), which encodes a zinc-finger transcription factor. The characterization of mutants showing a strong delay in *brlA* expression and conidiation led to the identification of genes *flbA-E, fluG* (Wieser et al., 1994; Wieser and Adams, 1995), and *tmpA* (Soid-Raggi et al., 2006), all required for *brlA* regulation. While *flbA-E* encode regulatory proteins (Lee and Adams, 1994, 1996; Yu et al., 1996; Arratia-Quijada et al., 2012; Herrero-Garcia et al., 2015), *fluG* and *tmpA* encode enzymes involved in the biosynthesis of independent signals needed for normal conidiation. *fluG* encodes a protein showing similarity to prokaryotic glutamine synthetase I (Lee and Adams, 1994) and is required for the production of an extracellular diorcinol-dehydroaustinol adduct (Rodríguez-Urra et al., 2012) that functions as conidiation signal. *tmpA* encodes a membrane oxido-reductase required for the synthesis of a FluG-independent extracellular conidiation signal not yet identified (Soid-Raggi et al., 2006).

Here we identify AfeA and TmpB adenylate-forming enzymes as novel proteins involved in the production of chemical signals that regulate asexual reproduction in *A. nidulans*. We show that AfeA is part of the previously identified TmpA pathway, while TmpB defines a novel conidiation-signaling pathway.

## Materials and methods

### Strains, media, transformation, and growth conditions

*Aspergillus nidulans* strains used in this work (Stringer et al., 1991; Kawasaki et al., 2002) are listed in Table S1. All strains were grown in 1% glucose supplemented minimal nitrate medium (Hill and Käfer, 2001). For diploid complementation tests two strains carrying different auxotrophic and conidial color markers were grown next to each other. After about 48 h small pieces of agar medium containing mycelia from both strains were transferred to MM to induce heterokaryon formation. Then conidia from heterokaryotic mycelia were collected and transferred to empty Petri plates and covered with warm agar-MM. Diploids formed homogenously growing colonies that produced larger, stable conidia able to form single colonies on MM. Developmental cultures (Aguirre, 1993) and nutrient starvation experiments (Skromne et al., 1995) were carried out as reported. Transformation was carried out by protoplast fusion (Yelton et al., 1984) or conidia electroporation (Sanchez and Aguirre, 1996; Sánchez et al., 1998). *fluG* overexpression and extracellular complementation experiments were performed as reported (Soid-Raggi et al., 2006).

### Genetics, β-galactosidase activity and microscopy

Crosses were performed using standard genetic techniques (Pontecorvo et al., 1953; Clutterbuck, 1969). β-galactosidase activity was determined as reported (Aguirre, 1993). Strains expressing fluorescence proteins GFP or BFP were imaged using an inverted Olympus FluoView™ FV1000 Confocal Microscope (Olympus, Japan) or a Leica DM6000 Confocal Microscope (Leica, Germany) both fitted with a laser with a GFP filter set to 488 nm excitation/505–550 nm emission and a BFP filter set to 385 nm excitation/450 nm emission. For FV1000 and Leica DM6000 oil immersion objectives 100X (1.42 NA) and 63X (1.4 NA) HCX PL APO CS were used, respectively. Confocal images were captured and evaluated using LAS AF or Olympus Fluoview software. For imaging in Olympus confocal microscopy, the “inverted agar block method” was used as previously described (Hickey et al., 2004), and the standard method for the Leica inverted confocal microscope. *In vivo* lipid body staining was carried out using BODIPY (4, 4-difluoro-3a, 4a,-diaza-s-indacene) 493/503 (Invitrogen) as reported (Zavala-Moreno et al., 2014), but without fixing the cells.

### Deletion of *afeA* and *tmpB* genes

The sequences of primers used in this work are listed in Table S2. To disrupt *afeA*, primers fluX27 and fluX28 carrying *SmaI* and *SalI* sites, respectively, were used to amplify an *afeA* 2.3 Kb fragment, using cosmid W19A10 as template. The PCR product was cloned into plasmid pCRII (Invitrogene) to generate pGS13. Plasmid pGS13 was digested with *SmaI* and *SalI* and the resultant fragment was cloned in BS(KSII), digested with the same enzymes, to produce pGS14. A *SphI-EcoRI* fragment, containg *A. nidulans argB* gene was obtained from plasmid pDC1 and subcloned into pGS14 to obtain pGS15, which was used to transform strain RMS011 by protoplast fusion. To delete *tmpB*, a 1501 bp 5′*tmpB* fragment was amplified with primers 1tmpAL and 2tmpAL. A 1618 bp 3′*tmpB* fragment was amplified with primers 3tmpAL and 4tmpAL. *A. fumigatus riboB* marker was amplified with primers 5riboB and 6riboB using plasmid pAfriboPstE1Skt(ssp1)-37 as template. The three PCR fragments were purified, mixed, and subjected to fusion PCR (Yu et al., 2004) using primers 7dtmpAL and 8dtmpAL. The final 5063 bp DNA fragment was used to transform strain A770. For the sequential deletion of *afeA* and *tmpA* genes, we first deleted *afeA* as follows. First, the 5′ and 3′ *afeA* PCR fragments were obtained using primers afeAFw1/pyrGafeARv2 and pyrGafeAFw3/afeARv4, respectively. *A. fumigatus pyrG* marker was amplified with primers pyrGforward and pyrGreverse, using plasmid PFNO3 as template. Finally, a 4800 bp *afeA–AfpyrG–afeA* deletion cassette was obtained by doble joint PCR using primers afeAFwNested7 and afeARvNested8 and used to transform strain 11035 by electroporation. Twenty PyrG^+^ transformants were obtained, and 10 were analyzed by PCR-restriction analyses to confirm *afeA* deletion and strain TOS100 was selected for further experiments. To delete *tmpA*, 5′ and 3′ regions were amplified using primers tmpAfw1/riboBtmpARev and riboBtmpAFw/tmpARev3, respectively. *A. fumigatus riboB* marker was amplified with primers 5Ribo and 6Ribo, using plasmid pAfriboPstE1Skt(ssp1)-37 as template. Primers tmpAFwNested and tmpARevNested were used to obtain a 5231 bp *tmpA*-ribo-*tmpA* fusion product, which was used to transform strain TOS100 by electroporation. 12 RiboB^+^ transformants were analyzed by Southern blot and the deletion of both genes was confirmed (not shown). Strain TOS200 was selected and crossed to strain CLK43 to get rid of the Δ*nkuA* mutation. Strain COS250 was selected for additional experiments.

### Tagging of AfeA, TmpA, and TmpB

For AfeA HA tagging, we first cloned *afeA* ORF into plasmid pJA7.1 2HA6H, which added a C-terminal tag of two copies of the hemaglutinin (HA) epitope and six consecutive histidine residues in plasmid pOS55. This plasmid was subjected to *in vitro* mutagenesis to produce plasmid pOS64, whose DNA sequence was confirmed. Transformation of strain PW-1 with pOS55 and pOS64 yielded 15 and 10 ArgB^+^ transformants, respectively. Four of the transformants obtained with pOS64 had a *fluffy* phenotype. Southern blot analysis showed that 2 transformants obtained with pOS55 (*afeA::HA*, wild type conidiation) and 4 with pOS64 (*AfeAK544N::HA, fluffy* phenotype) contained the expected plasmid integration events. Strains TOS10 (*afeA::HA*) and TOS16 (*AfeAK544N::HA*) were chosen for further analysis. To tag AfeA with GFP at its C-terminus, *afeA* 5′ and 3′ fragments were amplified with primers afeA5Fw/GFPafeARev and RiboafeA3/afeA4, respectively. *A. nidulans* codon-optimized GFP (Tan et al., 2014) was amplified with primers GFPFwnoATG and GFPNostopRv using plasmid pRPB614 as template. *AfriboB* marker was amplified with primers RiboFw and RiboRv and *afeA* ORF was amplified with GFPafeAFw and RiboafeARv primers. A 7700 bp fusion PCR product obtained with primers afeAFwNESTED7 and afeARvNESTED8 was used to transform strain 11035 by electroporation. Ten out of 27 transformants were analyzed by PCR and 8 generated the expected product. Strain TOR2-22 was chosen for further analysis. To label AfeA with red fluorescent protein mKate, the 5′ and the entire *afeA* ORF were amplified with primers afeA5′Fw and mKateafeARv. *A. nidulans* codon optimized mKate (Tan et al., 2014) was amplified with primers mKateFw and RibomKateRv from plasmid RPB616. *A. fumigatus riboB* marker was amplified with RiboFw and RiboRv primers. The 3′ fragment was amplified with primers RiboafeA3′ and afeA4. A 7636 bp fusion PCR product obtained with primers afeAFwNESTED7 and afeARvNESTED8 was used to transform *A. nidulans* strain 11035 by electroporation. Ten out of 36 transformants obtained were analyzed by PCR and strain TOS-70 was chosen for additional experiments.

To generate a TmpA::GFP construct, *afeA* 5′ fragment and entire *tmpA* ORF was amplified with primers tmpAGFPFw5′ and GFPtmpANoStopRev, and the 3′ fragment was amplified with primers pyrGstop/tmpAFw and tmpARv3. GFP was amplified with primers GFPnoATG and pyrGGFPRv from plasmid pRPB614 (Tan et al., 2014) and *AfpyrG* with primers pyrGFw and pyrGRev. A 8440 bp PCR product was obtained with primers tmpANESTED7 and tmpANESTED8 and used to transform *A. nidulans* strain 11035 by electroporation. Ten out of 11 transformants were analyzed by PCR and 6 generated the expected product. TOR3-11 strain was chosen for additional experiments.

Five PCR products were used to generate a TmpB N-terminal BFP construct. A 5′ fragment upstream the translation stop codon, was amplified with primers tmpB5′Fw and BFPtmpBRv. *A. nidulans* codon-optimized BFP was amplified with primers BFPFw and BFPnoStopRv using plasmid pRPB615A as template (Tan et al., 2014). *Af pyro4* marker was amplified with primers PyroFw and PyroRv, using plasmid PFNO3 as template (Nayak et al., 2006). Primers BFPtmpBFw, PyrotmpBRfv, PyrotmpB3′Fw and tmpB3′Rv were used to amplify *tmpB* ORF and *tmpB* 3′ fragment, respectively. Purified fragments were mixed and used in a fusion PCR with primers NestedtmpBFw and NestedtmpBRv. The 9363 bp BFP–tmpB-Afpyro4 cassette was used to transform *A. nidulans* strain 11035 by electroporation. Ten transformants were obtained and analyzed by PCR and 7 contained the expected integration event. Strain TOR1-8 was chosen for further analysis.

## Results

### The *afeA* gene is contiguous to *tmpA* and its inactivation also results in a *fluffy* phenotype

**Figure 1:**
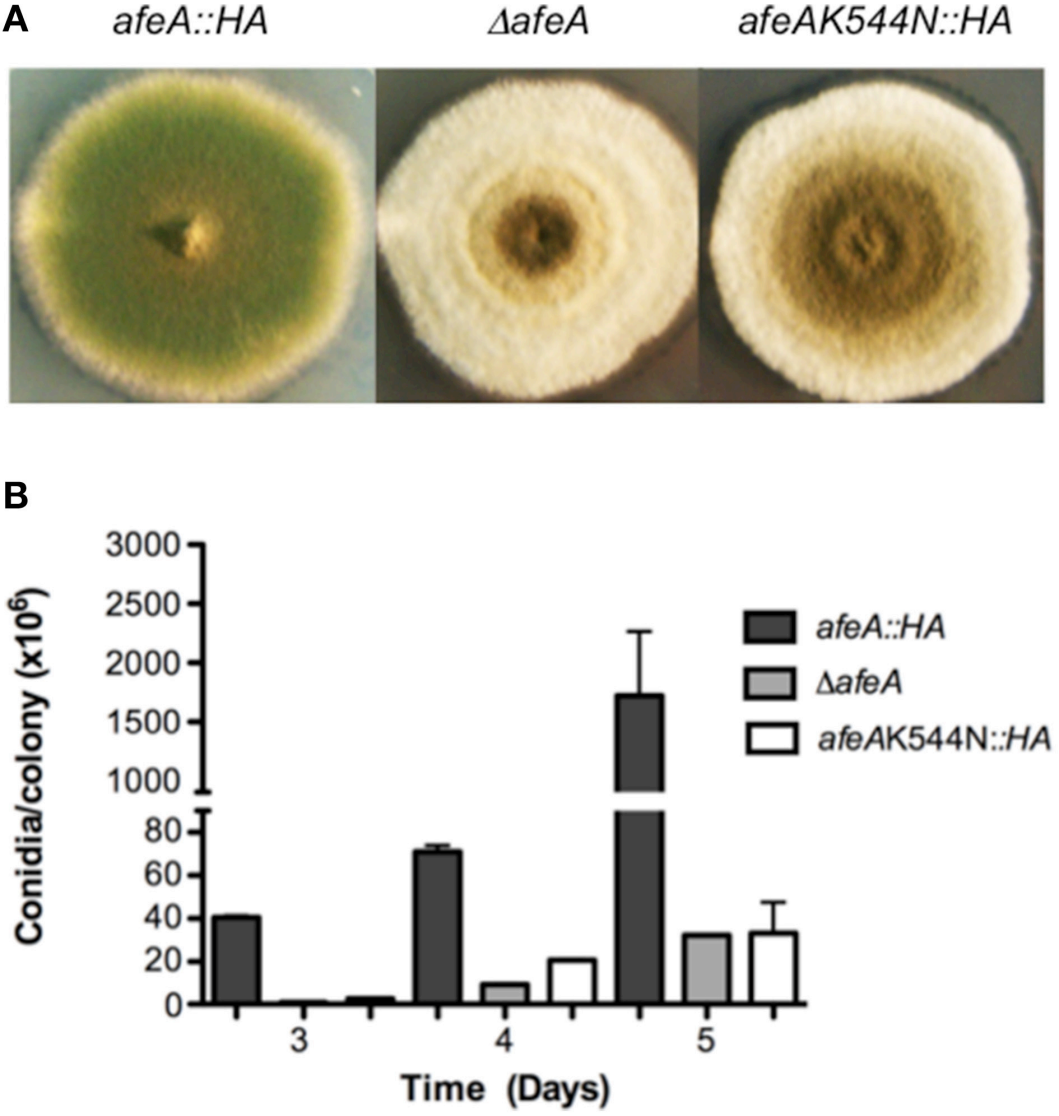
**Adenylate-forming enzyme AfeA is required for *A. nidulans* normal asexual development and contains a conserved lysine (K544) that is essential for its activity**. **(A)** Strains TOS10 (*afeA::HA*), CGS17 (Δ*afeA*), and TOS16 (*afeAK544N::HA)* were induced to conidiate and photographed after 5 days. **(B)** Strains from **(A)** were point inoculated, incubated for the indicated times and total conidia were harvested and counted. Error bars indicate standard deviation from three independent experiments.

We reported that inactivation of the *tmpA* gene, encoding a putative membrane oxidoreductase involved in the production of a sporulation signal, results in a *fluffy* phenotype (Soid-Raggi et al., 2006). *tmpA* (AN0055) and *afeA* (AN0054) genes were found located next to each other in an inverse orientation (Soid-Raggi et al., 2006), an arrangement that is conserved in several Aspergilli (http://www.aspergillusgenome.org/cgi-bin/locus.pl?locus=afeA&organism=A_nidulans_FGSC_A4; Arnaud et al., 2012). To determine if *tmpA* and *afeA* were functionally related, we generated plasmid pGS15 to inactivate the *afeA* gene by homologous recombination and evaluate its function. In pGS15, a 583-nucleotide *afeA* fragment encoding amino acids ^148^G to ^345^F was replaced by the *argB* gene, used as selective marker. This plasmid was used to transform *A. nidulans* strain RMS011 and 5 (TGS1-TGS5) out of 96 Arg^+^ transformants presented a *fluffy* cotton-like phenotype (Figure 1A), very similar to the one observed in Δ*tmpA* mutants (Soid-Raggi et al., 2006). When analyzed by Southern blot, 2 transformants had the hybridization pattern expected for the deletion of the *afeA* gene but showed additional bands, while transformants TGS1-TGS3 showed only the expected hybridization bands (Figure S1). Since *afeA* and *tmpA* deletion caused similar phenotypes and the two genes are contiguous in chromosome VIII, we wanted to exclude that the genetic replacement in *afeA* was affecting the expression of *tmpA* and *vice versa*. For this, we carried out diploid complementation tests (see Materials and Methods) between Δ*afeA* strains (TGS1-TGS5) and Δ*tmpA* mutant TGS6 (Soid-Raggi et al., 2006). In all cases the diploids obtained showed a wild-type phenotype (not shown), indicating that the deletion of one gene did not affect the expression of the other, and that both genes are individually required for normal asexual sporulation in *A. nidulans*.

Using Northern blot analysis, we detected a single *afeA* transcript, which accumulates at similar levels after 18 h of growth in liquid culture (0 h of development) and during asexual and sexual sporulation, and increases after 3 h of nitrogen starvation (Figures S2A,B). These results indicate that *afeA* and *tmpA* (Soid-Raggi et al., 2006) show similar patterns of mRNA accumulation.

### *afeA* encodes an AMP-binding protein related to plant 4CL-Lk enzymes

Originally, we sequenced the *afeA* gene (GenBank AY254381.2) and named it (adenylate forming enzyme A) after we found that it predicted a 583 amino acid protein containing an AMP-binding domain (PROSITE PS00455). AfeA is identical to protein An0054 in the AspGD database (http://www.aspgd.org), except that predicted An0054 contains two extra amino acids (MR) at its N-terminus. For clarity, AfeA amino acid residue numbering will be referred to An0054 585 amino acid protein. Although, the adenylate-forming superfamily includes a diverse set of enzymes using very different substrates, these enzymes are characterized by carrying out two-step reactions. In the first step ATP in the presence of Mg^*Z*+^ reacts with a carboxylate substrate to form an acyl-adenylate (acyl-AMP) intermediate with the release of pyrophosphate. In the second step this intermediate is transferred to an acceptor, in many cases CoA, releasing AMP. According to its carboxylate substrate, the members of this superfamily have been classified as acetyl:coA synthetases (AcCS), medium chain:coA synthetases (MCS), long chain:coA synthetases (LCS), 4-coumarate:CoA ligases (4CL), coumarate ligase like (4CL-Lk), luciferases, and the adenylation domains present in NRPSs.

AfeA shows higher similarity to plant 4CL and 4CL-Lk enzymes. 4CL is the third enzyme in the phenylpropanoid biosynthesis pathway in plants and forms coenzyme esters from hydroxycinnamic acids such as coumaric, caffeic, or ferulic acids (Hahlbrock and Grisebach, 1970). In contrast, 4CL-Lk function and substrate specificity are unknown in most cases. Figure S3 shows an alignment between AfeA and most similar proteins, including proteins whose activity or expression pattern is known. The phylogenetic analysis derived from this alignment (Figure S4) shows that 4CL and 4CL-Lk enzymes form two separate groups. AfeA groups in the 4CL-Lk clade along with homologs from other fungi such as *Aspergillus oryzae* (78% identity) and a bacterial enzyme, while plant 4CL enzymes form a different subgroup. However, enzymes acquire different substrate-specificity by mutation of a limited number of active site residues. Indeed, biochemical analysis of *A. thaliana At4g05160* and *At5g63380*, originally classified as 4CL-Lks by overall sequence similarity, showed that these enzymes correspond to fatty-acid CoA-ligases or LCS (Schneider et al., 2005).

Figure S3 also highlights several domains and motifs highly conserved in the adenylate-forming enzyme family (Conti et al., 1997). These include canonical residues ^449^R, ^455^K, ^457^K, and ^540^K. With different numbering, due to different protein length, these residues are conserved in AfeA. For example, being part of the highly conserved motif SGKI, canonical ^540^K corresponds to ^544^K in AfeA. Within box I, the motif SSGTTGLPKGV is highly conserved among 4CL and 4CL-Lks. In AfeA this region corresponds to TSGTGGLPKAA. The box II motif GEICIRG is a 4CL signature domain that in AfeA corresponds to GELYVRG. Notably, the central cysteine in this motif is replaced by aromatic amino acids tyrosine in AfeA and tryptophan in all 4CL-Lk enzymes. Of unknown function, N-terminal motif RSKLPDI is almost exclusive of 4CL enzymes (Cukovic et al., 2001) and is not well conserved in AfeA and its fungal orthologs. Likewise, Box L with a consensus sequence DRLK(D/E)L (Schneider et al., 2005), conserved in most 4CL and 4CL-Lks, corresponds to GRTKEL in AfeA and its *A. fumigatus* and *A. oryzae* orhologs.

As structures from 4CL-Lk enzymes have not been published, we tried to model AfeA using a 4CL from *Populus tomentosa* (Hu et al., 2010), which is 28% identical to AfeA. However, there were important discrepancies in the position of conserved regions between the 4CL crystal structure and the resulting model. A much better model was obtained using the Benzoate CoA ligase chain A (BCL) from *Burkholderia xenovorans* (Bains and Boulanger, 2007), which shares and overall identity of 21% with AfeA. A root-mean-square deviation (RMSD) value of 0.91 indicates a high level of overlap between BCL and AfeA model structures, as it can be appreciate in Figure S5A. The size of the binding pocket and the nature of the residues that line it determine the specificity of each adenylation domain. The size of the benzoate-binding pocket in this ligase shows a high degree of selectivity for this substrate and is lined by amino acids FAYIGSTHIK. This and the corresponding pocket in AfeA are hydrophobic and AfeA pocket is larger and lined by residues HLFWGMTVVK. AfeA residues ^357^G, ^359^T, and ^544^K show an orientation similar to the same residues in the BCL pocket. In AfeA predicted substrate binding pocket ^259^H, ^261^F, and ^364^V are in positions similar BCL ^236^F, ^238^Y, and ^332^I, respectively (Figure S5B). These results suggest that AfeA utilizes a hydrophobic substrate, larger than benzoate.

**Figure 2:**
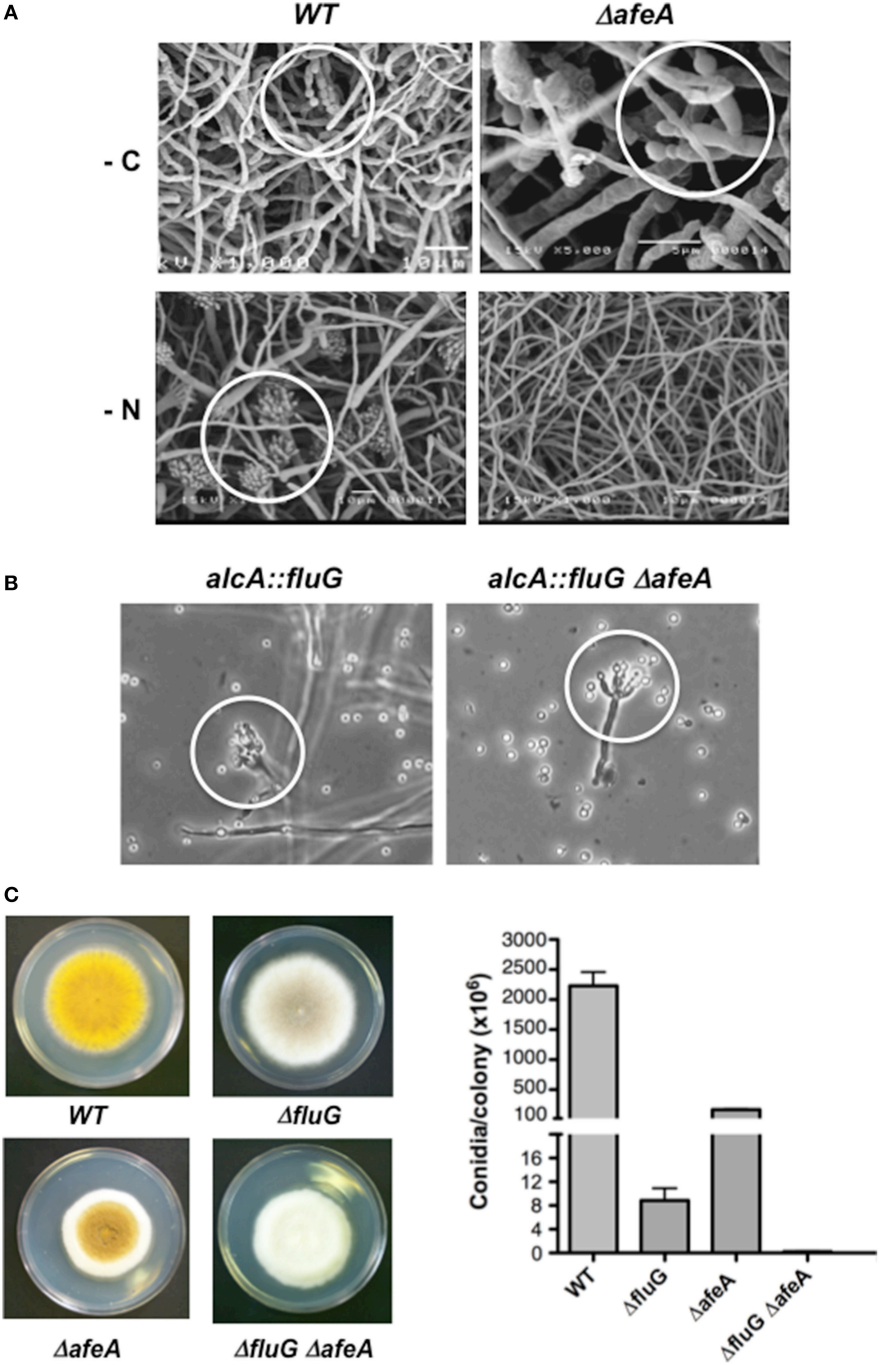
**The *afeA* gene is required for conidiation induced by nitrogen starvation and functions in a *fluG*-independent pathway. (A)** The wild type strain TJA22 (left panels) and Δ*afeA* mutant CGS17 (right panels) were grown for 18 h and shifted to media without carbon (-C) or without nitrogen (-N) for 20 h and samples were processed for electron scanning microscopy. White circles indicate morphologically reduced (-C) or fully developed conidiophores (-N). **(B)** Strains TBN68.11 (*alcA::fluG*) and CGS41 (*alcA::fluG* Δ*afeA*) were grown in MM-glucose for 14 h and then shifted to MM-threonine medium for 12 h to induce *fluG*, and samples were photographed under the microscope. White circles indicate morphologically reduced conidiophores. **(C)** Strains CLK43 (WT), CGS49 (Δ*fluG*), TGS1 (Δ*afeA*) and CGS34 (Δ*fluG* Δ*afeA*) were point inoculated, grown for 5 days and photographed, and the total number of spores per colony was counted. Bars represent standard deviation from three independent experiments.

**Figure 3:**
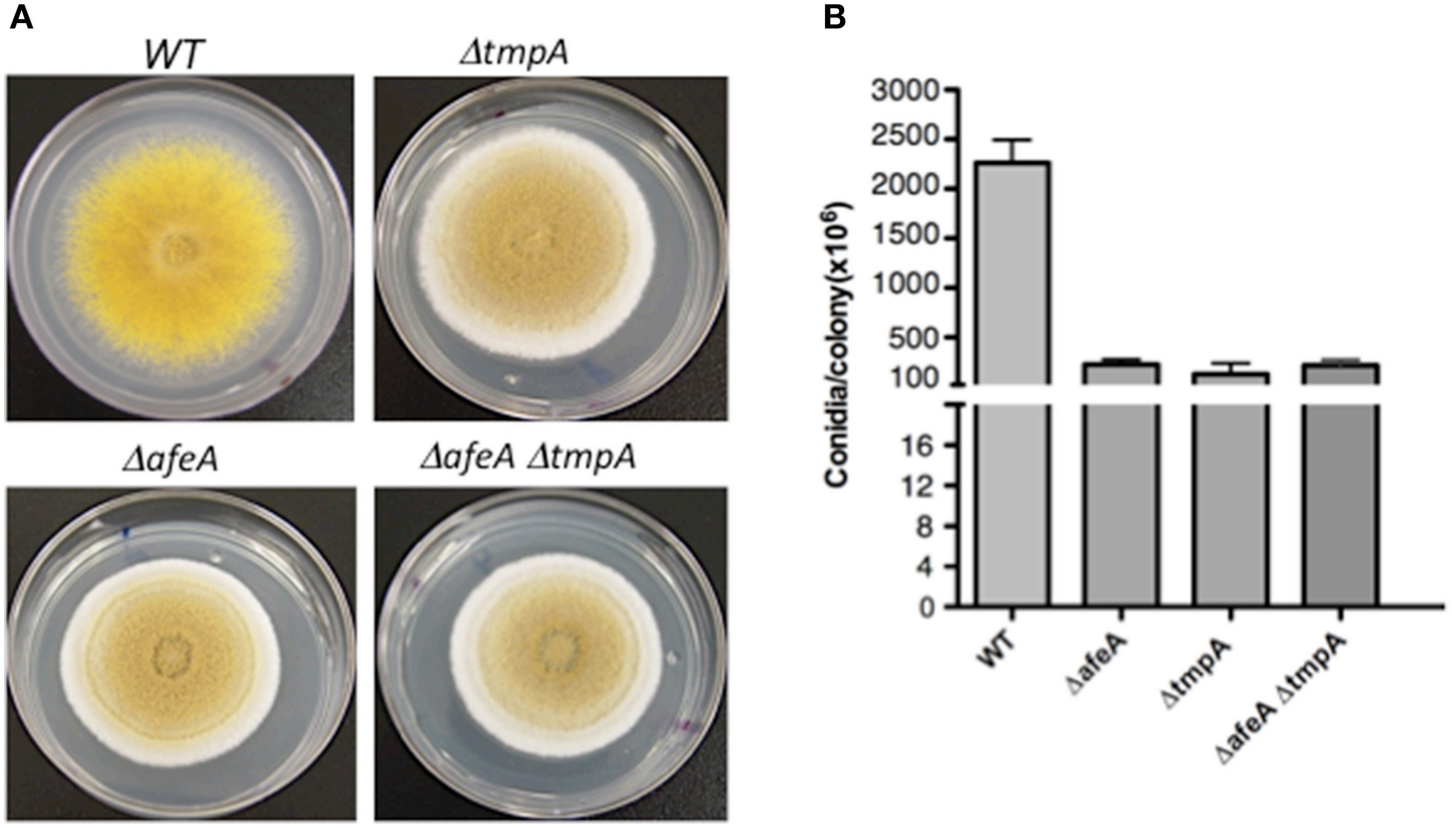
***afeA* and *tmpA* genes are part of the same regulatory pathway**. **(A)** Strains CLK43 (WT), TGS6 (Δ*tmpA*), TGS1 (Δ*afeA*), and COS250 (Δ*afeA* Δ*tmpA*) were point inoculated, grown for 5 days, photographed and the total number of spores per colony **(B)** were counted for two independent experiments.

^540^K is predicted to coordinate the carboxylate group at the adenylation active site in 4-coumarate:CoA ligases (Stuible et al., 2000). As shown in Figure S5B the equivalent lysine (^520^K) in *B. xenovorans* Benzoate CoA ligase interacting with the carboxylate of benzoate corresponds to ^544^K in our AfeA model. To evaluate AfeA ^544^K essential role in catalysis, we replaced it by N. To this end, we replaced the *afeA* gene with alleles producing either a wild type AfeA tagged with two copies of the hemaglutinin (HA) epitope and six consecutive histidine residues (AfeA::HA) or mutant AfeAK544N::HA, obtained by *in vitro* mutagenesis. Strains TOS10 (*afeA::HA*) and TOS16 (*AfeAK544N::HA*) were confirmed by Southern blot analysis (not shown) and DNA sequencing and chosen for further analysis. Western blot detection of AfeA tagged with the HA epitope showed that both transformants express similar levels of a ≈60 kDa protein, which was absent in Δ*afeA* (Figure S6) and WT strains (not shown). A strain expressing HA-tagged AfeA shows a morphology (Figure 1A) and conidiation levels similar to those seen in a wild type strain (see Figures 2C, 3B), whereas the strain expressing the mutant *AfeAK544N::HA* has a clear *fluffy* phenotype (Figure 1A) and conidiation levels similar to those observed in a Δ*afeA* mutant (Figure 1B). These results indicate that HA tagging did no alter AfeA function and that ^544^K is essential for AfeA activity, supporting its role as a *bona fide* adenylate-forming enzyme involved in asexual development.

### Like *tmpA*, the *afeA* gene is required for conidiation, *brlA* expression and the production of an extracellular sporulation signal different from the FluG factor

To evaluate *afeA* role in *brlA* expression we introduced the reporter *brlA::lacZ* (Aguirre, 1993) into Δ*afeA* strain TGS1 to obtain strain CGS17. Using this strain, we followed β-galactosidase activity in synchronous conidiating cultures exposed to air (Aguirre, 1993). After 25 h of induction of conidiation, β-galactosidase specific activity in Δ*afeA* strain CGS17 was about 20% of the one detected in the relevant WT strain TJA22 (Figure S2C). Likewise, when conidiation was induced by either carbon or nitrogen starvation (Skromne et al., 1995), *brlA::lacZ* expression in the Δ*afeA* mutant was about 50% of the one detected in the WT (Figure S2D). This last result is consistent with the fact that Δ*afeA* mutants, like Δ*tmpA* (Soid-Raggi et al., 2006) and other *fluffy* mutants (Arratia-Quijada et al., 2012), fail to sporulate under nitrogen starvation (Figure 2A).

Null mutants in the *fluG* gene show decreased conidiation because they fail to produce a chemical compound that function as sporulation signal, while *fluG* overexpression is enough to induce conidiation (Lee and Adams, 1994, 1996; Rodríguez-Urra et al., 2012). To determine if *afeA* was part of the *fluG*-pathway we used sexual crosses to introduce an *alcA::fluG* fusion (Lee and Adams, 1996), in which *fluG* is driven by the *alcA* gene inducible promoter, into a Δ*afeA* genetic background. As shown in Figure 2B, *fluG* overexpression induced conidiation in WT and Δ*afeA* genetic backgrounds, indicating that *afeA* is not required for *fluG* function. To further demonstrate that *afeA* belongs to a *fluG*-independent pathway we obtained a Δ*fluG* Δ*afeA* double mutant. As it can be observed in Figure 2C, a Δ*fluG* mutant shows a very *fluffy* phenotype but produces some spores in the center of the colony. A Δ*afeA* mutant is less *fluffy* and produces more spores than the Δ*fluG* mutant. However, the Δ*fluG* Δ*afeA* double mutant is *fluffier* that any of the single mutants and produced the lowest number of spores.

To demonstrate that *afeA* participates in the production of an extracellular factor that induces conidiation, we tested if Δ*afeA* mutants can conidiate when grown next to an *afeA*^+^ strain. Indeed, when a yellow-spore Δ*afeA* mutant was grown next to a green-spore Δ*fluG* mutant, a sporulation region of yellow and green conidiophores was formed between the two strains (Figure S7A). Such extracellular cross-complementation indicates that *afeA* is required for the production of an extracellular signal, different from the FluG factor. To further confirm this, a Millipore membrane with a pore size of 0.4 μm was used to separate a green-spore wild type strain from a yellow-spore Δ*afeA* strain, preventing any hyphal contact. As seen in Figure S7C, the Δ*afeA* mutant produced abundant yellow-spores under these conditions. In contrast, no sporulation was observed when the Millipore membrane separated two different Δ*afeA* mutants (Figures S7D–F). In a different type of experiment, liquid media in which a wild type or a Δ*afeA* strain was grown for 24 h was filtered through 0.22 μm Millipore membranes and inoculated with spores from a Δ*afeA* mutant. As is observed in Figures S7D–F, only the medium used by the wild type strain induced Δ*afeA* sporulation.

### AfeA and TmpA act in the same pathway

As Δ*afeA* and Δ*tmpA* mutants show virtually identical phenotypes and there was no extracellular cross-complementation between them (not shown), this suggested that both genes acted in a single pathway. To demonstrate this, we generated Δ*afeA* Δ*tmpA* double mutants. As both genes are contiguous and the *fluffy* mutant phenotypes are undistinguishable, it was difficult to obtain double mutants by sexual crosses. Instead, we carried out the sequential deletion of both genes. Δ*afeA* strain TOS100 was used to delete *tmpA*, using *AfriboB* gene as selective marker. Twelve RiboB^+^ transformants were analyzed by Southern blot and the deletion of both genes was confirmed (not shown). Single and double mutants displayed the same colony morphology and produced very similar conidiation levels (Figure 3), indicating that *afeA* and *tmpA* act on the same conidiation pathway.

### AfeA is localized in discrete globular bodies that co-localize with lipid bodies and the plasma membrane, and its overexpression results in conidiation

The phylogenetic analysis of the adenylate forming enzyme family in *A. thaliana* showed that in contrast to 4CL enzymes, all 4CL-Lk proteins contain a clear peroxisome localization signal (Shockey et al., 2003). AfeA lacks a PTS1 peroxisome signal, but it could contain PTS2 or Pex19BS peroxisome targeting signals. AfeA *in silico* analysis using Phobius suggests that it is a non-cytoplasmic protein that contains two hydrophobic regions, while the program Target Predictor detects putative Pex19BS (LVRTLITGLKAh; amino acids 61–72 with a Block *E*-value of 0.55) and PTS2 (RgdcVLvHL; amino acids 76–84 with a Block *E*-value of 0.061) peroxisome targeting signals in AfeA.

**Figure 4:**
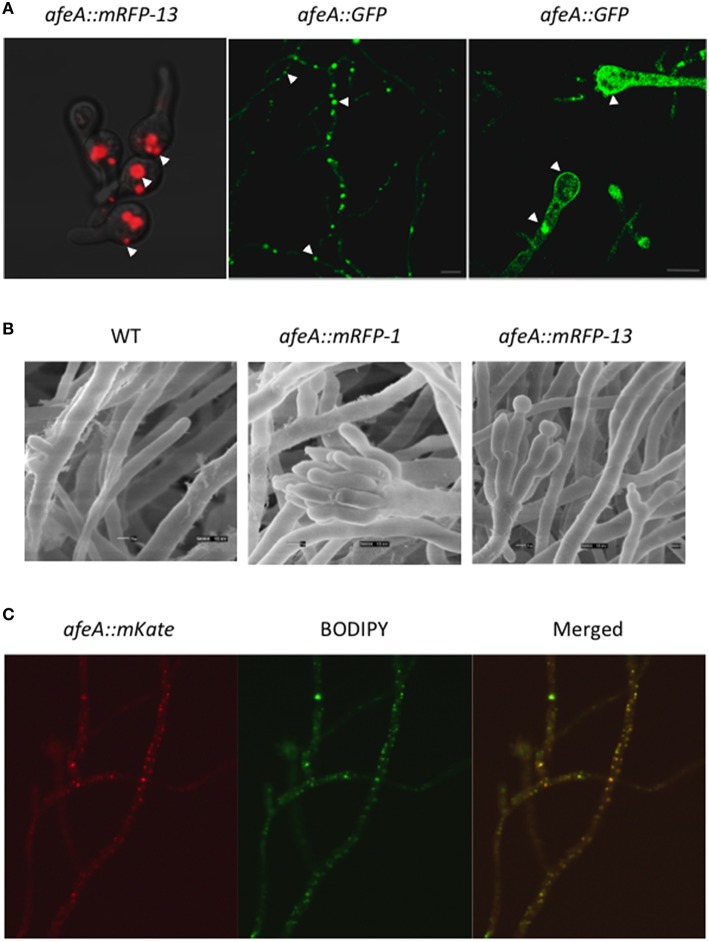
**AfeA is localized in discrete globular bodies and the plasma membrane and its overexpression results in conidiation. (A)** Strain TOS330 (*afeA::mRFP13*) was inoculated in MM-glucose liquid medium, grown for 12 h and shifted to MM-threonine medium for 3 h to induce the *alcA* promoter and samples were photographed using confocal microscopy (left panel). Strain TOR2-22 (*AfeA::GFP*) was grown for 20 h on solid medium and mycelium (central panel) and conidiophore (right panel) structures were observed using a Leica inverted confocal microscope. Arrows indicate cytoplasmic globular structures and the plasma membrane. **(B)** Conidia (1 × 10^6^ per ml) from strains PW-1 (wild type), TOS320 (*afeA::mRFP11*), and TOS330 (*afeA::mRFP13*) were grown MM-glucose medium for 14 h and then transferred to MM-threonine, to induce the *alcA* promoter, and grown for 18 h. Samples were fixed and observed by electron scanning microscopy. **(C)** Strain TOS-70 (*afeA::mKate*) was grown in MM for 12 h without shaking and shifted to MM without nitrogen for 3 h. After this, samples were stained with BODIPY (see Materials and Methods) and photographed using confocal microscopy.

To experimentally determine AfeA localization, plasmid pafeAmRFP containing an inducible AfeA C-terminal mRFP fusion was generated by PCR and *in vitro* recombination (Toews et al., 2004). This plasmid was used to transform strain PW-1 and Southern Blot analyzis of the 12 ArgB^+^ transformants obtained showed 3 transformants with a hybridization pattern consistent with plasmid integration at the *argB* locus (not shown), and strain TOS330 was chosen for further analysis. To detect AfeA::mRFP, conidia from TOS330 strain were germinated on glass coverslips submerged in liquid medium, incubated for 12 h at 37°C and then transferred to minimal-threonine medium for 3 h to induce the *alcA* promoter. As shown in Figure 4A (left panel), AfeA::mRFP shows a very discrete distribution in germlings, being localized in organelle-type bodies of heterogeneous size.

To determine if this *AfeA::mRFP* fusion was functional and if AfeA overexpression was enough to induce conidiation, we grew strain TOS330 during 14 h in shaken culture and transferred mycelia to threonine medium for 18 h. Samples were fixed and observed by electron scanning microscopy. Under these conditions the wild type strain PW-1 did not conidiate. In constrast, strains TOS320 (*afeA::mRFP-1*) and TOS330 (*afeA::mRFP-13*) produced conidiophores with metulae, phialides, and conidia (Figure 4B). These results show that AfeA C-tagged with mRFP is functional, that afeA overexpression induces asexual sporulation and support AfeA localization in discrete bodies during conidial germination.

To further confirm that AfeA C-tagging did not affect its function and validate its localization, we generated a new fusion in which AfeA was tagged with GFP and expressed from its own promoter. Strain 11035 was transformed with a construct based on the *AfriboB* selective marker. Fifteen transformants were obtained; 5 were confirmed by PCR analysis (not shown) and strains TOR2-3 and TOR2-22 were selected for characterization. These strains, containing the GFP construct at the endogenous *afeA* locus, conidiated as the wild type strain (not shown), confirming that GFP tagging did not interfere with AfeA function. When mycelia from strain TOR2-22 was used to localize AfeA::GFP, an intense fluorescent signal was detected again in discrete bodies and a less intense signal was detected at the plasma membrane (Figure 4A, central panel). A similar pattern was observed in young conidiophores, although a more evenly distributed signal was also observed in some conidiophores (Figure 4A, right panel). These results indicate that AfeA is a non-cytoplasmic protein that localizes in round bodies of heterogeneous size and at the plasma membrane. Since these organelle-type bodies seemed too large and heterogeneous to be peroxisomes, we tested if they corresponded to lipid-bodies, as these are heterogeneous in size and number, and contain lipid-metabolizing enzymes (Pu et al., 2011). For this purpose we used wild type conidiating strain TOS-70, which expresses AfeA labeled with red fluorescent protein mKate from its own promoter, and stained it with the molecular probe BODIPY 493/503, which is specific for lipid bodies and produces a green florescent signal when bound to these structures. Because of the low fluorescence signal we observed with *afeA::mKate* and based of *afeA* induction by nitrogen starvation (Figure S2B), we used a 3 h nitrogen starvation treatment to increase *afeA::mKate* expression. Figure 4C shows hyphae in which AfeA::mKate red signal is localized in discrete points of different sizes and BODIPY staining of the same hypha produced a green signal with very similar pattern, which can be better appreciated in the merged image. Therefore, it can be concluded that AfeA is found in lipid bodies and at the plasma membrane.

### TmpB, a TmpA homolog, defines a novel sporulation pathway

Having defined the *afeA-tmpA* conidiation pathway, we wanted to know whether the larger *tmpA* homolog identified previously (Soid-Raggi et al., 2006), played any role in this pathway. This *tmpA* homolog corresponds to gene AN9129 (Galagan et al., 2005), which we have renamed here as *tmpB*. This gene, located on chromosome VI, encodes a protein of 989 amino acids. As AfeA, TmpB corresponds to an adenylate-forming enzyme. However, it contains the additional TmpA-like oxidoreductase domain at its C-terminus. An alignment between AfeA and TmpB AMP-domains shows that overall similarity is very low (not shown). In contrast, the oxidoreductase domain is 55% similar to TmpA. As indicated before (Soid-Raggi et al., 2006), TmpB adenylation domain shares high similarity to adenylation domains (A) of bacterial non-ribosomal peptide synthetases (NRPS) and fungal NPS12-type NRPSs. Indeed, TmpB architecture (adenylation plus a ferric reductase domain) is shared by members of the mono-bimodular ChNPS12/ETP NRPS family, whose origin is suggested to predate the divergence of eubacteria and fungi (Bushley and Turgeon, 2010). As TmpB A domain shares a relatively high similarity to the phenylalanine activating subunit of the gramicidin synthetase 1 (PheA) from *Bacillus brevis*, we used PheA crystal structure (Conti et al., 1997) to model TmpB adenylation domain. As shown in Figure S10A, there is a good overlap between PheA and TmpB model structures (RMSD of 1.31). PheA ^235^D, ^324^G, and ^330^I are well placed to form hydrogen bonds with the α-amino group of phenylalanine and the aspartic acid is conserved in all NRPSs. The α-carboxylate of phenylalanine is stabilized by an electrostatic interaction with the invariant ^517^K from the C-terminal domain. A conserved aromatic residue (F, W, or H), proposed to play a key role in the positioning of α-amino and α-carboxylate groups (Lee et al., 2015) corresponds to ^239^W in PheA. These critical residues are conserved (^238^D, ^309^G, ^315^I, ^242^W, and ^495^K) in TmpB and have a very similar orientation in the TmpB model (Figure S10B). In addition, residues ^322^A and ^307^C have a similar orientation between PheA and TmpB, respectively. These results support TmpB role as a NRPS in which the A domain would be involved in the activation of an amino acid substrate, while the TmpA domain might be involved in oxidation of either the substrate or the activated substrate. There are other genes located next to *tmpB*, including a putative enoyl-coA hydratase/isomerase gene, which are conserved among several Aspergilli, suggesting that *tmpB* is part of a gene cluster involved in secondary metabolism.

To delete *tmpB* we used a 5063 bp DNA fragment, generated by double-joint PCR (Yu et al., 2004), to transform strain A770 (Nayak et al., 2006). Six RiboB^+^ transformants were obtained and subject to Southern blot analysis. Two transformants contained the expected deletion event (Figure S8), and strain TΔTMPB4 was chosen for further analysis. *tmpB* deletion resulted in a clear *fluffy* phenotype in the center of the colony, while the rest of the colony appeared to sporulated normally. When the total number of spores per colony was counted, the Δ*tmpB* mutant produced about 1.8 times less conidia than the wild type strain (Figure S9A). However and in contrast to the *afeA* gene, which is strictly required for nitrogen starvation-induced conidiation, *tmpB* was dispensable to conidiate in response to carbon or nitrogen starvation (Figure S9B).

**Figure 5:**
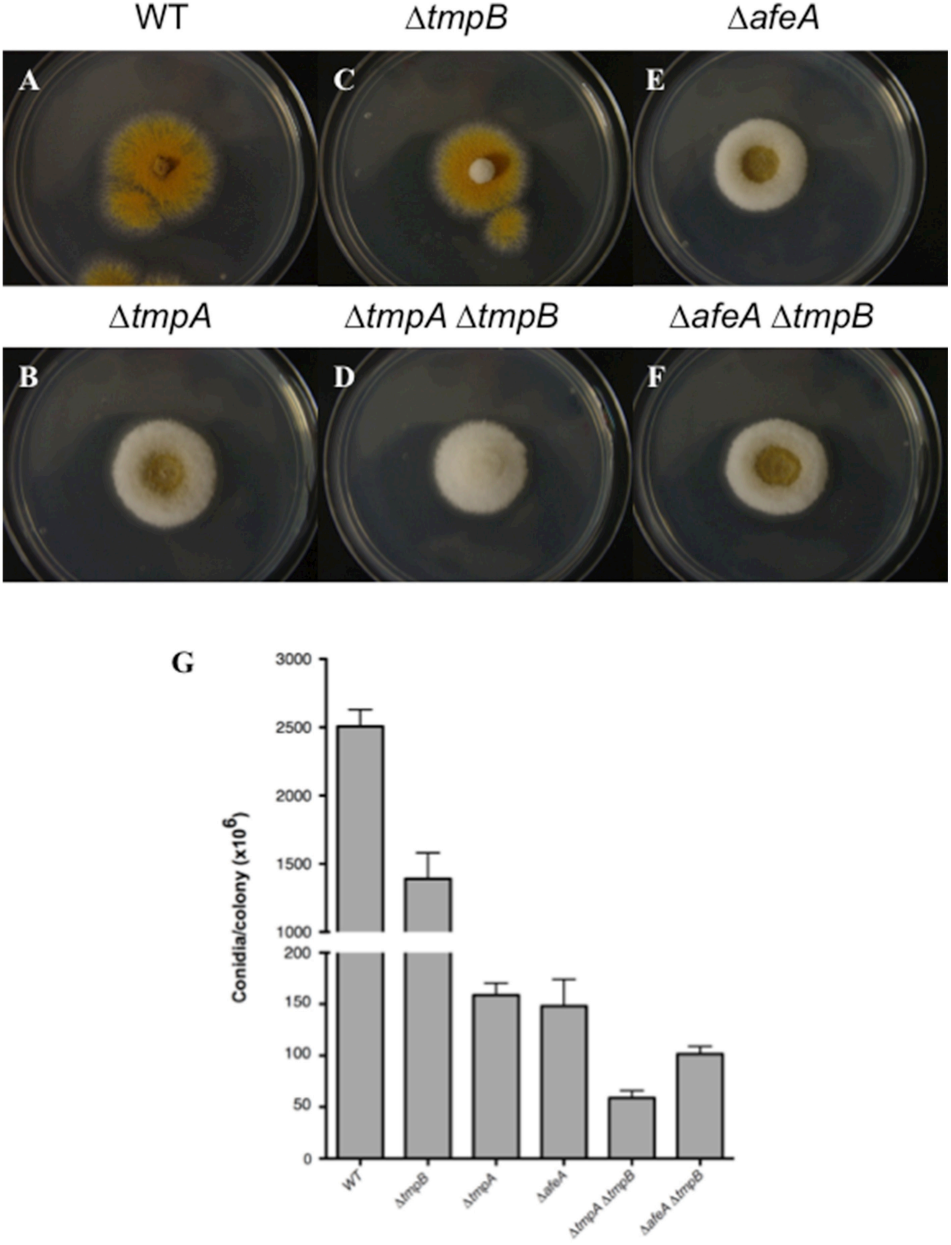
**TmpB is a TmpA homolog that defines an independent sporulation regulatory pathway. (A–F)** Strains CLK43 (WT), CΔTMPBP1 (Δ*tmpB*), TGS1 (Δ*afeA*), TGS6 (Δ*tmpA*), CΔTMPAB3 (Δ*tmpA* Δ*tmpB*), and CΔTMPBAFEA4 (Δ*afeA* Δ*tmpB*) were point inoculated, incubated during 3 days and photographed. **(G)** The total number of spores per colony was counted after 5 days.

To determine *tmpB* interactions with *afeA* and *tmpA*, we generated double mutants and compared the morphology and conidiation levels. As seen in Figure 5C, *tmpB* mutant fluffy morphology in the center of the colony is contrasting with *tmpA* and *afeA* mutants (Figures 5B,E) which at this time of growth show sporulation at the center of the colony and a cotton-like appearance at the periphery. This suggests that AfeA-TmpA and TmpB are involved in the production of different compounds that regulate sporulation during colony development. Indeed, double Δ*tmpA* Δ*tmpB* mutants failed to sporulate in the center as well as in the periphery of the colony (Figure 5D). This was not the case for Δ*afeA* Δ*tmpB* double mutants (Figure 5F), which showed some sporulation in the center of the colony. However, when conidia were counted it became clear that although the Δ*afeA* Δ*tmpB* produced about twice as much conidia when compared to the Δ*tmpA* Δ*tmpB* mutant, it conidiated about 50% less than single Δ*tmpA* or Δ*tmpB* mutants (Figure 5G). These results suggest the possibility that the oxidoreductase activity of TmpA and the TmpA-domain in TmpB could substitute each other in the oxidation of chemical signals generated either by AfeA or TmpB A domain, and the lack of both activities results in lack of both signals. However, it is unclear why some sporulation still occurs in the center of the colony in the absence of both AfeA and TmpB. Perhaps, the absence of both enzymes results in the induction of an alternative sporulation pathway. In any case, TmpB defines a novel sporulation regulatory pathway, different from the FluG and AfeA-TmpA conidiation pathways.

**Figure 6:**
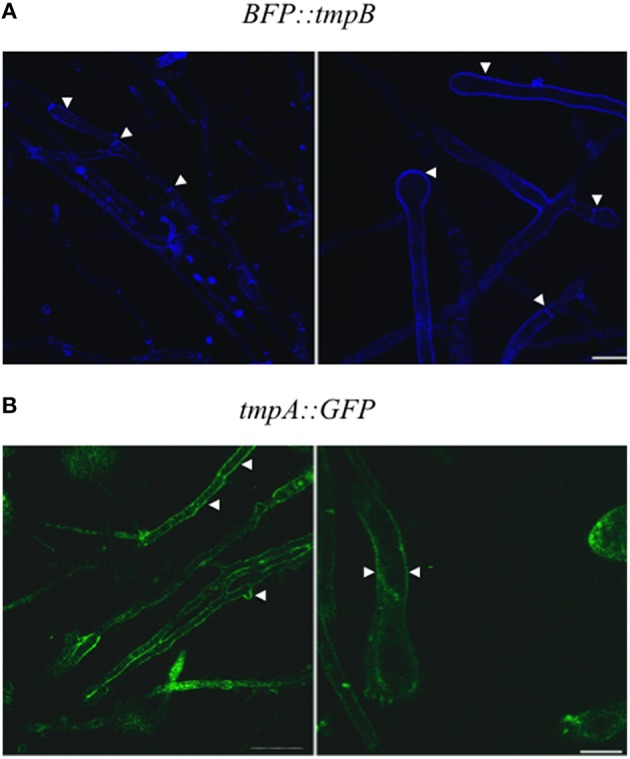
**TmpB and TmpA are localized at the plasma membrane. (A)** Strain TOR1-8 (*BFP::tmpB*) was grown for 20 h on solid medium and mycelium (left panels) and conidiophore (right panels) structures were observed using a Leica inverted confocal microscope. **(B)** Strain TOR3-11 (*tmpA::GFP*) was grown and observed as in **(A)**. Arrowheads indicate the plasma membrane and septa in mycelium, and plasma membrane in conidiophores.

To determine TmpB localization, a PCR construct in which TmpB was tagged at its N-terminus with *A. nidulans* codon-optimized BFP was used to transform *A. nidulans* strain 11035 by electroporation. Of 10 transformants analyzed by PCR, 7 contained the expected integration event and strain TOR1-8 was chosen for further analysis. As TOR1-8 showed normal conidiation, this indicated that the *BFP::tmpB* fusion is functional. In mycelia, *BFP::tmpB* was localized at the plasma membrane and septa and in conidiophores it was localized at the plasma membrane (Figure 6A). Using a TmpA::GFP fusion, we previously localized this protein at the plasma membrane of germinating conidia. However, the fusion was partially functional and we were unable to detect the protein in mycelia or conidiophores. To confirm that both TmpB and TmpA were plasma membrane proteins, we generated a new PCR TmpA::GFP construct containing a glycine spacer. The PCR product was used to transform strain 11035 by electroporation. Ten out of 11 transformants were analyzed by PCR and 6 generated the expected product. Strain TOR3-11, showing normal conidiation, was chosen for additional experiments. Like TmpB, in hyphae TmpA was localized in septa and the plasma membrane, and in conidiophores in the plasma membrane (Figure 6B).

## Discussion

### The role of the AfeA-TmpA pathway in the regulation of asexual reproduction

We have shown that AfeA and TmpA are enzymes that participate in a single pathway involved in the biosynthesis of a yet unknown chemical signal that regulates cell communication, *brlA* expression and asexual reproduction in *A. nidulans*. As L-phenylalanine ammonia-lyase (PAL) enzyme activity and PAL-encoding genes have been detected in fungi, including *A. nidulans* (Hyun et al., 2011), AfeA relationship to 4CL and 4CL-Lk enzymes might indicate the presence of a phenylpropanoid-like metabolism in fungi. The phenylpropanoid pathway is responsible for the biosynthesis of numerous secondary metabolites, several involved in plant signaling (Dixon and Paiva, 1995).

4CL and firefly luciferases utilize hydrophobic cyclic compounds and contain regions sharing similarity to the fatty acyl-coA synthetases (FACS) signature motif DGWLHTGDIGXWXPXGXLKIIDRKK (Black et al., 1997). However, 4CL and luciferases contain an aspartate or a lysine instead of the highly conserved glycine a position 16, and a leucine instead of a lysine at position 24. The equivalent region in AfeA is 24 amino acid-long (QGWFRTGDVAYVNDGLYYIVGRTK) and depending on the gap position, shows a conserved glycine at position 15 (16) and a threonine at position 24, suggesting that catalytically AfeA is more related to FACS than to 4CL or luciferase. Moreover, AfeA is more closely related to the peroxisomal 4Cl-Lk enzyme At4g05160 (Figure S4), which shows the catalytic capacity to activate medium-chain fatty acids, carrying or not a phenyl substitution, long-chain fatty acids, and the capacity to contribute to jasmonic acid biosynthesis by initiating the β-oxidation of its precursors (Schneider et al., 2005). The 12 amino acid residues identified to form the substrate-binding pocket in At4CL2 correspond to VFAWGGAGGGIV in At4g05160, while the corresponding region in AfeA is LFLYGAGLGGVV. Notably, both regions are highly hydrophobic and among 4CL and other 4CL-LK enzymes, only At4g05160 (W) and AfeA (F) have an aromatic amino acid at position 4. Therefore, AfeA might display catalytic properties similar to those found in At4g05160. Jasmonic acid is a classical plant oxylipin also detected in several fungi (Cole et al., 2014) and, when added exogenously, it can affect fungal metabolism for example by inhibiting aflatoxin production (Goodrich-Tanrikulu et al., 1995). Although, treatment of Δ*afeA* and Δ*tmpA* mutants with filters containing 1, 5, 10, and 100 μM methyl jasmonic acid did not suppress their conidiation defects (not shown), the possibility that AfeA participates in the biosynthesis of a jasmonic acid-type metabolite cannot be ruled out.

Another 4CL-Lk, the non-peroxisomal enzyme ACOS5, has also been shown to use hydroxylated medium- or long-chain fatty acids to generate the corresponding acyl-coA esters (de Azevedo Souza et al., 2009). These are then used by type III polyketide synthases to catalyze condensation with malonyl-coAs and yield triketide and tetraketide alfa-pyrones, required for pollen development and sporopollenin biosynthesis in *A. thaliana* (Kim et al., 2010). It is clear that in *A. nidulans* and other fungi acetyl-coA and malonyl-coA are precursors of diverse polyketides and terpenes (Thines et al., 2006; Lim and Keller, 2014) and that PKSs participate in the synthesis of chemical signals that regulate conidiation (Márquez-Fernández et al., 2007).

At4g05160 and ACOS5 are peroxisomal and non-peroxisomal enzymes, respectively. AfeA does not contain a clear peroxisome-targeting signal and as we have shown here it is found in lipid bodies and the plasma membrane, which is consistent with the presence of two hydrophobic regions in AfeA and the presence of lipid metabolizing hydrophobic enzymes in lipid bodies (Pu et al., 2011). Notably, the putative fatty acid dioxygenase PpoA, required for the biosynthesis of oxylipins involved in *A. nidulans* sexual and sexual development, is also localized in lipid bodies (Tsitsigiannis and Keller, 2007). The localization of AfeA in lipid bodies, the induction of *afeA* by nitrogen starvation (Figure S2B), and the fact that nitrogen starvation induces lipid body accumulation in diverse fungi, including *Ustilago maydis* (Zavala-Moreno et al., 2014), offer additional support to AfeA connection to lipid metabolism.

### TmpB defines a novel condiation regulatory pathway

TmpB belongs to the mono-bimodular ChNPS12/ETP NRPS family, from which very little is known in fungi and only the *Alternaria brassicicola* and *A. fumigatus* orthologs, called TmpL, have been studied in some detail. Kim et al. (2009) confirmed our prediction about TmpL homologs being flavin-binding proteins (Soid-Raggi et al., 2006) and showed that TmpL inactivation in *A. brassicicola* led to the production of less pigmented conidia displaying age-related deterioration signs and increased sensitivity to H_2_O_2_, as compared to a WT strain. Likewise, deletion of *tmpL* in *A. fumigatus* resulted in the production of conidia with abnormal subcellular morphology and increased sensitivity to H_2_O_2_. Notably, loss of TmpL function resulted in avirulence in both, plant and animal pathogens. Ectopic expression of a TmpL::GFP fusion in *A. brassicicola* showed that it was not expressed in mycelia while in conidia it was detected in a punctuated pattern, consistent with TmpL localization in peroxisomes and Woronin bodies. The lack of TmpL in this fungus also led to an increased expression of antioxidant genes and the nuclear localization of a GFP-Yap1 fusion in conidia, while yap1 overexpression led to partial suppression of the *tmpL* mutant phenotype. Based on this, it was proposed that TmpL regulates redox homeostasis and virulence in these fungi (Kim et al., 2009).

In comparison, *A. nidulans* conidia from Δ*tmpB* mutants do not show decreased viability and are not sensitive to oxidative stress (H_2_O_2_ and menadione; not shown). *tmpB* (AN9129) is expressed during growth in liquid medium (Andersen et al., 2013), as well as during conidiation in solid medium (Garzia et al., 2013) and consistent with this, we showed that TmpB is localized at the plasma membrane of growing hyphae and conidiophore structures. Based on our results, we hypothesize that TmpB A domain is involved in the adenylation of a hydrophobic amino acid, and that the oxidoreductase domain is involved in the oxidation of the acylated product. Whether the TmpB product itself would be a sporulation signal or the substrate of another NRPS is to be determined.

### Self-self communication, colony development, and reproduction

It is clear that microbial cells can from communities with organized patterns of cell types, and a fungal colony represents a clear example of such developmental pattern. In an air interphase, *A. nidulans* conidia germinate and form hyphae that grow and branch radially for a period of about 20 h, before they become competent to respond to induction of conidiation. After this period, colonies produce conidiophores as they grow and only hyphae at the border of the colony do not produce conidiophores and continue to grow (reviewed in Noble and Andrianopoulos, 2013). Since substrate availability is heterogeneous during colony development, being partially degraded at the center and unexplored at the periphery of the colony, *A. nidulans* colony development strategy simultaneously allows conidiation and substrate colonization, before nutrients are exhausted. Indeed, trancriptional profiling during colony development in *Aspergillus niger* (Levin et al., 2007) and *Neurospora crassa* (Kasuga and Glass, 2008) shows that gene expression profiles are clearly different at the center and the periphery of the colony. However, very little is known about the mechanisms that regulate such patterns. Our results indicate that AfeA-TmpA and TmpB pathways are involved in the production of different compounds that regulate sporulation during colony development, as TmpB is required for sporulation at the center of the colony and AfeA-TmpA is needed for conidiation at the periphery of the colony (Figure 5). Suggested substrates for AfeA and TmpB might help to identify their products in the near future and determine if the AfeA-TmpA, TmpB, and FluG signals form gradients across *A. nidulans* colonies and if there is any interplay among them.

Nutrient starvation is a primary stimulus to induce microbial sporulation. *A. nidulans* carbon starvation in liquid culture results in a rapid and high induction of the *brlA* gene and the differentiation of minimal conidiophores, while nitrogen starvation causes a gradual induction of *brlA* and the formation of fully differentiated conidiophores (Skromne et al., 1995). Null *fluffy flbB, flbC, flbD, flbE, afeA*, and *tmpA* but not *tmpB* mutants conidiate well under carbon starvation but fail to conidiate under nitrogen starvation (Soid-Raggi et al., 2006; Arratia-Quijada et al., 2012). This suggests that both, FluG and AfeA-TmpA (but not TmpB) signals are required for the developmental response to nitrogen starvation, as well as the existence of alternative pathways to induce conidiation in response to carbon starvation.

## Author note

We recently found that AfeA overexpression requires TmpA for conidiation to take place, indicating that TmpA functions downstream of AfeA.

## Author contributions

JA designed experiments, wrote the MS, obtained funding. GS performed and designed experiments, contributed to MS writing. OS performed and designed experiments, contributed to MS writing. JR performed and designed experiments, contributed to MS writing.

### Conflict of interest statement

The authors declare that the research was conducted in the absence of any commercial or financial relationships that could be construed as a potential conflict of interest.
