# Supplementary material for: The Adenylate-Forming Enzymes AfeA and TmpB Are Involved in Aspergillus nidulans Self-Communication during Asexual Development
Source: Front Microbiol. 2016 Mar 23;7:353. doi: 10.3389/fmicb.2016.00353 (PMC4804170; doi:10.3389/fmicb.2016.00353)
Supplement: Supplementary file 1 [file DataSheet1.docx]

**Table S1.** *Aspergillus nidulans* strains used in this study

| Strain | Genotype | Source |
| --- | --- | --- |
| RMS011 | *pabaA1, yA2; ΔargB::trpCΔB; trpC801, veA1* | ([Stringer et al., 1991](#_ENREF_47)) |
| TGS1-TGS5 | *pabaA1, yA2, ΔargB::trpCΔB; trpC801, ΔafeA::argB, veA1* | This work, RMS011 transformed with pGS15 |
| BL001 | *pabaA1, yA2; ΔargB::trpCΔB; trpC801, ΔtmpA::pDC1, veA1* | ([Sanchez et al., 1998](#_ENREF_42);[Soid-Raggi et al., 2006](#_ENREF_46)) |
| MH1164 | *yA2, suadE20, adeE20; amdS-Δ223; amdA7; riboB2, veA1* | M. Hynes |
| CGS1-CGS2 | *biA1; ΔargB::trpcCB; metG1; trpC801, ΔtmpA::pDC1, veA1* | This work, progeny from BL001 X MH1164 |
| CGS3 | *biA1, ΔargB::trpCΔB; riboB, trpC801, ΔtmpA::pDC1, veA1* | This work, progeny from BL001 X MH1164 |
| CGS5 | *biA1, ΔargB::trpCΔB; metG1; riboB, trpC801, ΔtmpA::pDC1, veA1* | This work, progeny from BL001 X MH1164 |
| TGS6 | *pabaA1, yA2, ΔargB::trpCΔB; trpC801, ΔtmpA::argB, veA1* | ([Soid-Raggi et al., 2006](#_ENREF_46)) |
| TJA22 | *biA1; metG1; brlA::lacZ::argB, veA1* | ([Aguirre, 1993](#_ENREF_2)) |
| CGS17 | *biA1; brlA::lacZ::argB, ΔargB::trpcΔB; metG1; trpC801, ΔafeA::argB, veA1* | This work, progeny from TGS1 X TJA22 |
| TBN68.11 | *pabaA1, yA2; alcA(p)::ΔN(1-423)fluG::trpC; veA1* | T. Adams |
| CGS41 | *pabaA1, yA2; alcA(p)::ΔN(1-423)fluG::trpC; ΔafeA::argB, veA1* | This work, progeny from TBN68.11 X CGS17 |
| RBN119 | *biA1, yA2; ΔfluG, argB2;metG1; veA1* | ([Wieser and Adams, 1995](#_ENREF_56)) |
| CGS34 | *pabaA1, yA2; ΔfluG::trpC; ΔafeA::argB, veA1* | This work, progeny from RBN119 X TGS1 |
| CGS49 | *pabaA1, yA2; ΔfluG::trpC; veA1* | ([Soid-Raggi et al., 2006](#_ENREF_46)) |
| CLK43 | *pabaA1, yA2; veA1* | ([Kawasaki et al., 2002](#_ENREF_26)) |
| FGSC26 | *biA1; veA1* | Fungal Genetics Stock Center |
| CΔTMPBP1 | *pabaA1, yA2; ΔtmpB::riboB* | Progeny from TΔTMPB4 x CDS2 |
| TΔTMPB4 | *pyrG89; pabaB22, ΔtmpB::riboB; riboB2* | A770 transformed with cdtmpB-AfriboB |
| CDS2 | *cfwA2, biA1, riboB2, pyroA4, paba, yA2* | D. Schnabel |
| CΔTMPAB3 | *pabaA1, yA2;ΔtmpA::argB; ΔtmpB::riboB* | Progeny from TΔTMPB4 X TGS6 |
| CΔTMPBAFEA4 | *pabaA1, yA2, ΔafeA::argB; ΔtmpB::riboB; veA1** | Progeny from TGS1 X TΔTMPB1 |
| 11035 | *pyrG89; pyroA4; ΔnkuA::argB; riboB2; veA1* | ([Nayak et al., 2006](#_ENREF_36)) |
| A770 | *pyrG89; pabaB22; riboB2* | FGSC |
| PW-1 | *biA1, argB2; methG1, veA1* | P. Weglenski |
| TOS10 | *biA1, argB2; methG1, afeA::2HA6H::argB; veA1* | This work, PW-1 transformed with plasmid pOS55 |
| TOS16 | *biA1, argB2; methG1, afeAK542N::2HA6H::argB; veA1* | This work, PW-1 transformed with plasmid pOS64 |
| TOS100 | *pyrG89; pyroA4; riboB2; ΔafeA:: AfpyrG; ∆nkuA::bar; veA1* | This work, 11035 transformed with PCR construct afeA-AfpyrG-afeA |
| TOS200 | *pyrG89; pyroA4; riboB2; ΔafeA::AfpyrG; ΔtmpA::AfriboB; ∆nkuA::bar; veA1* | This work, TOS100 transformed with PCR construct ΔtmpA-AfriboB-tmpA |
| COS250 | *pabaA1, yA2; ΔafeA::AfpyrG; ΔtmpA::AfriboB; ∆nkuA::bar; veA1* | This work, progeny from TOS200 X CLK43 |
| TOS320 | *biA1; metG1; argB::alcA::afeA::mRFP; veA1* | This work, PW-1 transformed with plasmid pafeAmRFP |
| TOS330 | *biA1; metG1; argB::alcA::afeA::mRFP; veA1* | This work, PW-1 transformed with plasmid pafeAmRFP |
| TOR2-22 | *pyrG89; pyroA4; riboB2; GFP::afeA:: AfriboB; ∆nkuA::bar; veA1* | This work, 11035 transformed with PCR construct GFP-afeA-AfriboB |
| TOR3-11 | *pyrG89; pyroA4; riboB2; tmpA::GFP::AfpyrG; ∆nkuA::bar; veA1* | This work, 11035 transformed with PCR construct tmpA-GFP-AfpyrG |
| TOR1-8 | *pyrG89; pyroA4; riboB2; BFP::tmpB:: Afpyro; ∆nkuA::bar; veA1* | This work, 11035 transformed with PCR construct BFP-tmpB-Afpyro |
|  |  |  |
| TOS-70 | *pyrG89; pyroA4; riboB2; afeA::mKate::AfriboB; ∆nkuA::bar; veA1* | This work, 11035 transformed with PCR construct afeA-mKate-AfriboB |
|  |  |  |

*It may contain *ΔnkuA::argB*

**Table S2.** DNA primers used in this study

| Primer | Sequence (5’ to 3’) |
| --- | --- |
| fluX27 | ATACCCGGGAGCAAATCAAAAAGAAG |
| fluX28 | ACTGTCGACAATGGGTCTGTGGGAGTG |
| fluX19 | ACTATTGCCACTTCTGC |
| afeANotI | GTTTAGCGGCCGCCTACGGCAATACGCCCTCCATGCATAAT |
| NotIafeA | GTTTAGCGGCCGCATGGGATCGAATGCACGGAGTCAGCCT |
| afeAK/N(SspI)F | GTACGGCAAGCGGGAATATTCAGCGCTTCAAGC |
| afeAK/N(SspI)R | GCTTGAAGCGCTGAATATTCCCGCTTGCCGTAC |
| afeA1 | CACCATGGGTCTGTGGGAGTCG |
| afeA2 | TACGGCAATACGCCCTCC |
| 1TMPAL | CTGTGTGGTATGTACGGATGG |
| 2TMPAL | ATTTTAATCCCATGTGATCAAACGAGCCAGAAGGTAAGACGTATATGC GCGG |
| 3TMPAL | CGTCACACTCATGTAACGGTTCTGCAGCGCAGGCGAAGTCTTGAGGCT GCTGG |
| 4TMPAL | ACTCCTGCCGAAAAGACCGTGC |
| 5RiboB | CTGGCTCGTTTGATCACATGG |
| 6RiboB | GCGCTGCAGAACCGTTACATG |
| 7DTMPAL | AGCGTATTTCCTTGGCACCTTC |
| 8DTMPAL | GGTGTTTTAGAGTCGTAGGGGG |
| afeA5’Fw | CCTCTGCAAATAGCTTCT |
| GFPafeARv | CAATTCTTCACCTCCTGACATGAGTTCGTCGAATAAT |
| GFFwNoATG  GFPRvNoStop  GFPafeAFw  RiboafeARv  RiboFw  RiboRv  RiboafeA3’  afeA4 | TCAGGAGGTGAAGAATTGTTCGC  GCGAGAGCCTGTGGAGTGGAACC  GGTTCCACAGGCTCTCGCAGAATGGGTCTGTGGGAG  TGTGATCAAACGAGCCAGTCATACGGCAATACGCCC  CTGGCTCGTTTGATCACATGG  GCGCTGCAGAACCGTTACATG  GTAACGGTTCTGCAGCGCTACGACGCTGGTTTTCGT  TCCCAGCAATAAGACACC |
| tmpAGFPFw5’ | GCTTGCGTTCAAGAACATACGGA |
| GFPtmpARev | AACAATTCTTCACCTCCTGACATGGCTCTTGAATGTTTCGAGAGCT |
| GFPnoATG  GFPnostopRv  GFPtmpAFw  pyrGtmpARv  pyrGFw  pyrGRev  pyrGtmpAFw  tmpA3’Rv  tmpANESTED7  tmpANESTED8 | TCAGGAGGTGAAGAATTGTTCGC  CGCAGAGCCTGTGGAACCGGGAC  GTCCCGGTTCCACAGGCTCTCGCTCTCAGCCCGAAGAGGTTATCGT  GGGTGAAGAGCATTGTTTGAGGCTCATGAATCAAATATTGGC CCAT  CGTCTGTAAACGAAGTCTCCCAG  GGTTCTGATCCACAGACG  CGCATCAGTGCCTCCTCTCAGACTTTCCGATGCATCACTTACGCTG  CGTCTGTAAACGAAGTCTCCCAG  GGTTCTGATCCACAGACG  TGACAAACTGGAACTTCC |
| tmpB5’Fw | CTCTGCAAATAGCTTCTTTGGCC |
| BFPtmpBRv | TTTTCCTTAATAAGCTCTGACATGACGATGACAGTGTTCTTCGTAG |
| BFPFw  BFPnoStopRv  BFPtmpBFw  PyrotmpBRfv  PyroFw  PyroRv  PyrotmpB3’Fw  tmpB3’Rv  NestedtmpBFw  NestedtmpBRv | ATGTCAGAGCTTATTAAGGAAAA  GAGAACCTGGAGTGGGCGGTGGAACGTGATCCGGTACTTCCTGGTC  GACCAGGAAGTACCGGATCACGTTCCACCGCCCACTCCAGGTTCTC  TAGTAATCCAGCATCTGATGTCCCTAACTGTCCCAGATCGCCCCGT  GGACATCAGATGCTGGATTACTA  GCGAGTGTCTACATAATGAAGGA  TCCTTCATTATGTAGACACTCGCACGACGGCGAAGTCTTGAGGCG  CGTGTCCTCTCGCGCACCTCCTC  GCAAACTAAGCCCAAGCTGT  CCGAACGCCATCTACAATGC |
| mKateafeARv | AATAAGTTCGGAGACCATTACGGCAATACGCCCTCC |
| mKateFw | ATGGTCTCCGAACTTATT |
| RibomKateRv | TGTGATCAAACGAGCCAGCTATCGATGACCAAGTTT |
| afeAFwNESTED7 | GATACTCCGAACTCCAGC |
| afeARvNESTED8 | TCCCAGCAATAAGACACC |
