# Supplementary material for: The Adenylate-Forming Enzymes AfeA and TmpB Are Involved in Aspergillus nidulans Self-Communication during Asexual Development
Source: Front Microbiol. 2016 Mar 23;7:353. doi: 10.3389/fmicb.2016.00353 (PMC4804170; doi:10.3389/fmicb.2016.00353)
Supplement: Supplementary file 2 [file Image1.pdf]

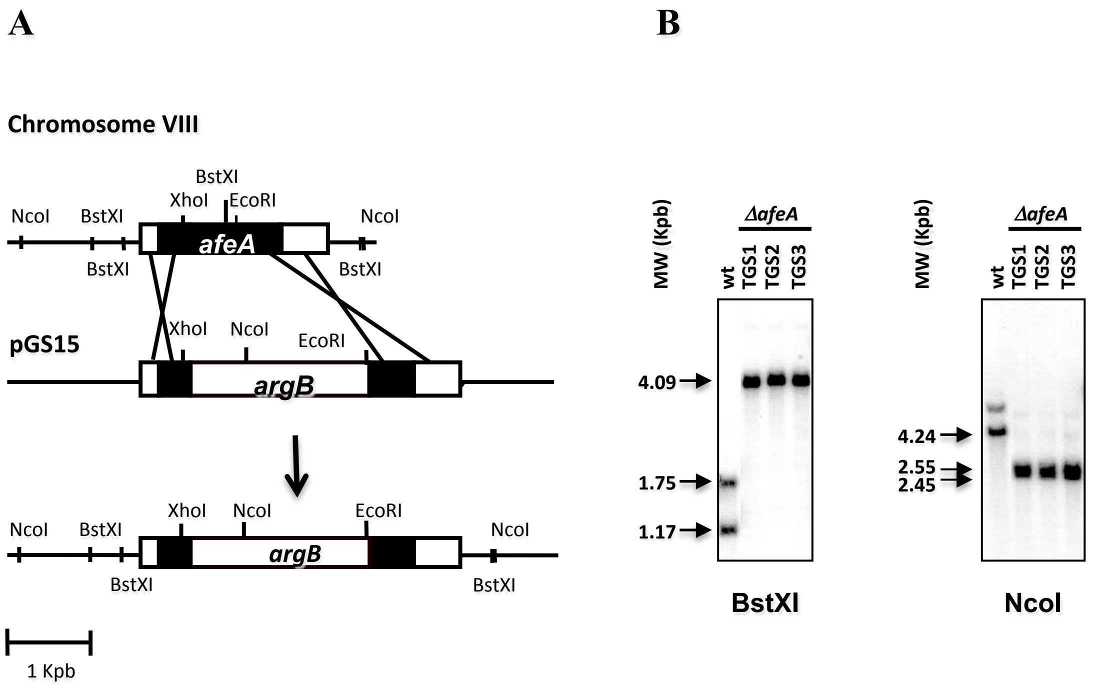

Figure S1. Disruption of the *afeA* gene. **(A)** Plasmid pGS15 was constructed by replacing a 591 pb region of *afeA* by the *argB* gene, used as selective marker. The eliminated AfeA region goes from 148G to 345F. pGS15 was linearized and used to transform strain RMS011. **(B)** Total DNA extracted from strains RMS011 and TGS1-3 (*fluffy* phenotype) was digested with the indicated restriction enzymes and used for Southern blot analysis, using a *SmaI-XbaI* fragment from plasmid pGS13 which corresponds to *afeA* ORF. Hybridization patterns are consistent with the integration event illustrated in **(A)**.
