# Supplementary material for: The Adenylate-Forming Enzymes AfeA and TmpB Are Involved in Aspergillus nidulans Self-Communication during Asexual Development
Source: Front Microbiol. 2016 Mar 23;7:353. doi: 10.3389/fmicb.2016.00353 (PMC4804170; doi:10.3389/fmicb.2016.00353)
Supplement: Supplementary file 3 [file Image2.pdf]

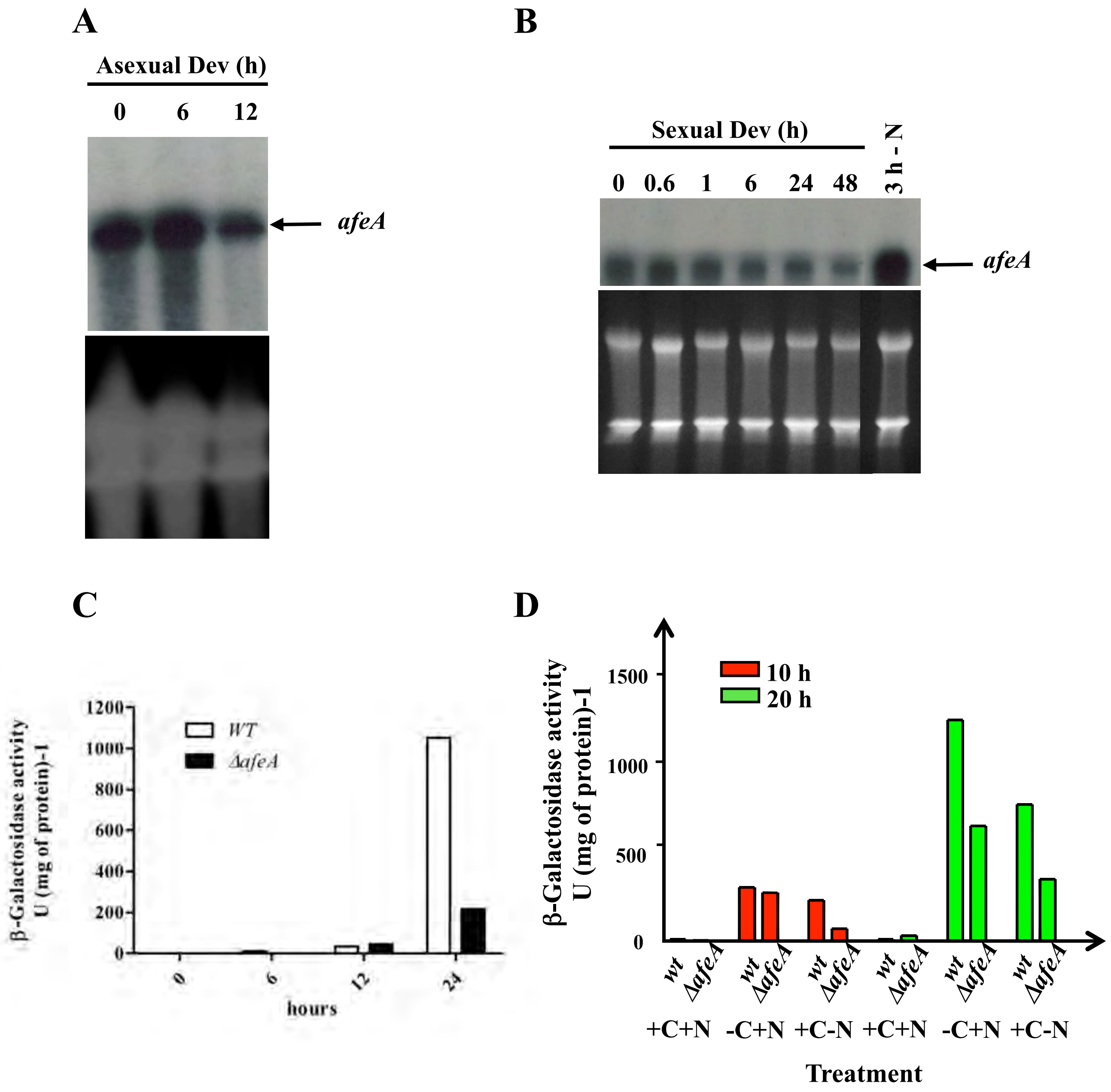

Figure S2. *afeA* and *brlA* gene expression during growth, asexual and sexual development, and nutrient starvation. **(A)** Strain TJA22 was grown for 18 h in liquid medium (0 h) and mycelia was filtered and exposed to air to induce asexual development. Samples were processed for RNA extraction and used for Northern blot analysis using an *afeA*-specific probe. **(B)** WT strain CLK43 was induced to undergo sexual development in confluent plates or transferred to medium lacking nitrogen (–N) for 3 h. At the indicated time points samples were collected and processed for RNA extraction and Northern blot analysis using an *afeA*-specific probe. Lower panels in **A** and **B** show ribosomal RNA as loading controls. **(C-D)** Strains TJA22 **(C)** and CGS17 *(ΔafeA*) carrying a *brlA::lacZ* fusion were grown for 18 h (0 h) and induced to conidiate or transferred to minimal medium with (+C+N) or without carbon (-C+N), or without nitrogen (+C-N) **(D).** Samples harvested at the indicated time points were used to determine β-galactosidase specific activity.
