# Supplementary material for: The Adenylate-Forming Enzymes AfeA and TmpB Are Involved in Aspergillus nidulans Self-Communication during Asexual Development
Source: Front Microbiol. 2016 Mar 23;7:353. doi: 10.3389/fmicb.2016.00353 (PMC4804170; doi:10.3389/fmicb.2016.00353)
Supplement: Supplementary file 4 [file Image3.pdf]

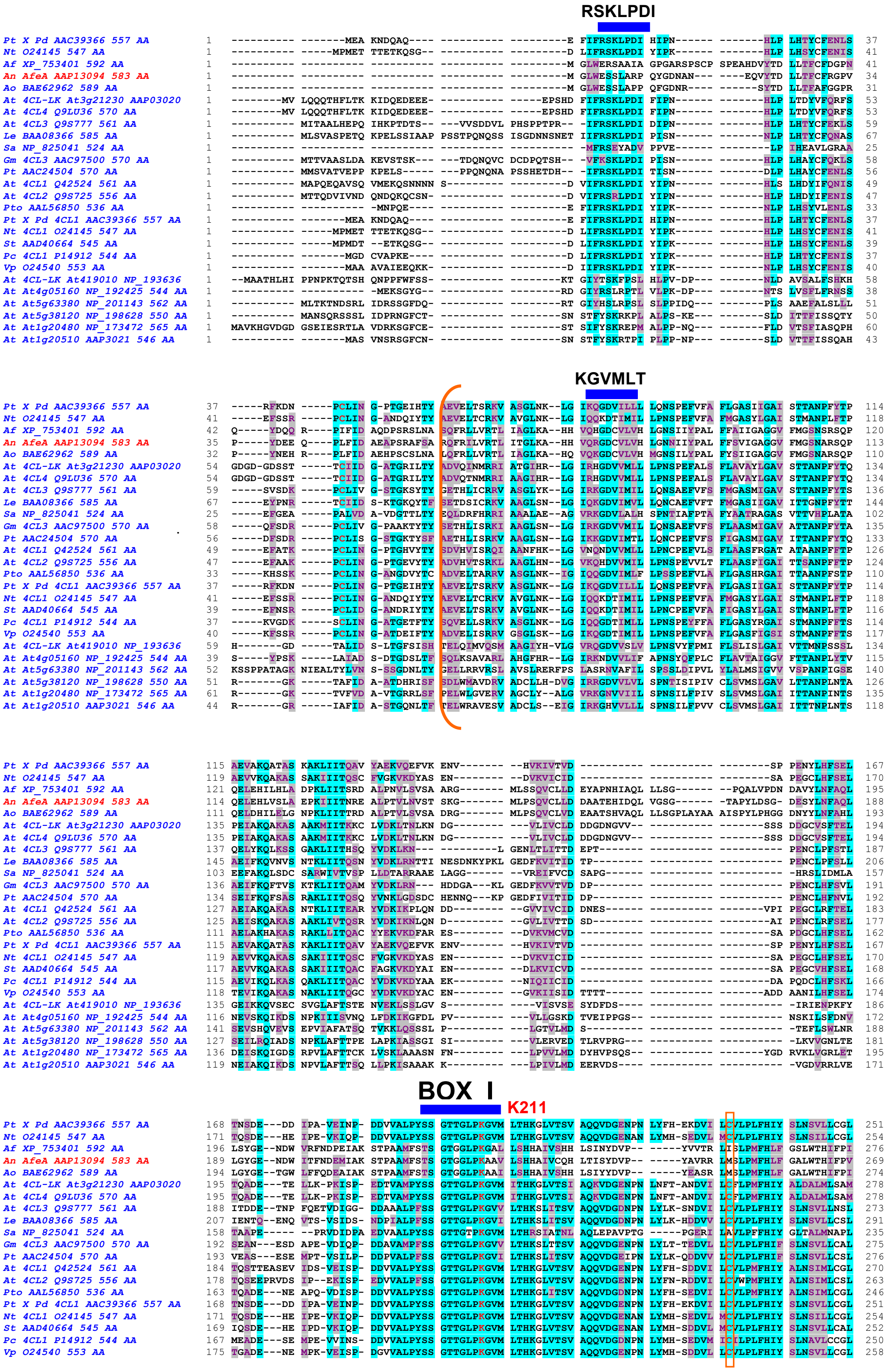
RSKLPDI
KGVMLT
BOX I
K211

```
At 4CL-LK At419010 NP_193636    187 SIMKE--SFG FVPKPLIKQ- DDVAAIMYSS GTTGASKGVL LTHRNLIASM ELFVRFEASQ YEYPGSSNVY LAALPLCHIY GLSLFVMGLL  273
At At4g05160 NP_192425 544 AA   173 MELSE--PVS EYPFVEIKQ- SDTAALLYSS GTTGTSKGVE LTHGNFIAAS LMVTMDQDLM G---EYHGVF LCFLPMFHVF GLAVITYSQL  256
At At5g63380 NP_201143 562 AA   189 SDSS----SV NPFQVQVNQ- SDPAAILFSS GTTGRVKGVL LTHRNLIAST AVSHQRTLQD PVN--YDRVG LFSLPLFHVF GFMMMIR-AI  270
At At5g38120 NP_198628 550 AA   182 MMKKE--PSG QAVRNQVHK- DDTAMLLYSS GTTGRSKGVN SSHGNLIAHV ARYIAEPFEQ -----PQQTF ICTVPLFHTF GLLNFVLATL  263
At At1g20480 NP_173472 565 AA   196 MIETE--PSE SRVKQRVNQ- DDTAALLYSS GTTGTSKGVM LSHRNLIALV QAYRARFG-- -----LEQRT ICTIPMCHIF GFGGFATGLI  275
At At1g20510 AAP3021 546 AA      172 MMKKE--PSG NRVKERVDQ- DDTATLLYSS GTTGMSKGVI SSHRNLIAMV QTIVNRFGSD --D--GEQRF ICTVPMFHIY GLTAFATGLL  254

                                                           293
Pt X Pd AAC39366 557 AA         252 RVGSAILLMQ KFEIVTLMEL VQKYKVTIAP FVPPVVLAVA K--CPVVDKY DLSSIRTVMS GAAPMGKELE DTVRAKLP-N AKLGQGYGMT  338
Nt O24145 547 AA                 255 RVGAAILIMQ KFDIAPFLEL IQKYKVSIGP FVPPIVLAIA K--SPIVDSY DLSSVRTVMS GAAPLGKELE DAVRTKFP-N AKLGQGYGMT  341
Af XP_753401 592 AA             277 RYGQPLFVLP RFELTQYVAA IYHYQITETY MVPAMIHVFN R--SAPPIAD YMRTLRYVGV AGAPIDGPSM EQFQRLLHPH ARATQLWGMT  364
An AfeA AAP13094 583 AA         270 RYGQPLYVLS RFEISRFVAA VYQYQITETY MVPAMIHAFN R--CTLPLAD YFGSLRYVGV AGAPIDGASM QQFREFLPLD ADASQLWGMT  357
Ao BAE62962 589 AA              275 RYGQPLYILP RFDIAQYVAA VYQYQITEAY MVPAMIHAFN R--CAFPVAD YLQSLRYVGV AGAPIDGASM QQFQELLHVD ANASQLWGMT  362
At 4CL-LK At3g21230 AAP03020    279 RTGAALLIVP RFELNLVMEL IQRYKVTVVP VAPPVVLAFI K--SPETERY DLSSVRIMLS GAATLKKELE DAVRLKFP-N AIFGQGYGMT  365
At 4CL4 Q9LU36 570 AA           279 RTGAALLIVP RFELNLVMEL IQRYKVTVVP VAPPVVLAFI K--SPETERY DLSSVRIMLS GAATLKKELE DAVRLKFP-N AIFGQGYGMT  365
At 4CL3 Q9S777 561 AA           274 RSGATVLLMH KFEIGALLDL IQRHRVTIAA LVPPLVIALA K--NPTVNSY DLSSVRFVLS GAAPLGKELQ DSLRRRLP-Q AILGQGYGMT  360
Le BAA08366 585 AA              292 RAGAAVLIMQ KFEIGALLEL IQSHRVSVAA VVPPLVLALA K--NPMVDKY DLSSIRVVLS GAAPLGRELE LALLNRVP-H AIFGQGYGMT  378
Sa NP_825041 524 AA              236 RLGATVVVLP RFDLDTFLAA IEKHRITHLY VAPPIVLALA K--HPAVAQY DLSSLKYVIS AAAPLDADTA AACSRRLG-V PPVGQAYGMT  322
Gm 4CL3 AAC97500 570 AA          276 RAGSAVLLMQ KFEIGTLLEL IQRHRVSVAM VVPPLVLALA K--NPMVADF DLSSIRLVLS GAAPLGKELV EALRNRVP-Q AVLGQGYGMT  362
Pt AAC24504 570 AA              277 RAGSAVLLMQ KFEIGSLLEL IQKHNVSVAA VVPPLVLALA K--NPLEANF DLSSIRVVLS GAAPLGKELE DALRSRVP-Q AILGQGYGMT  363
At 4CL1 Q42524 561 AA           271 RVGAAILIMP KFEINLLLEL IQRCKVTVAP MVPPIVLAIA K--SSETEKY DLSSIRVVKS GAAPLGKELE DAVNAKFP-N AKLGQGYGMT  357
At 4CL2 Q9S725 556 AA           264 RIGATILIMP KFEITLLLEQ IQRCKVTVAM VVPPIVLAIA K--SPETEKY DLSSVRMVKS GAAPLGKELE DAISAKFP-N AKLGQGYGMT  350
Pto AAL56850 536 AA             247 RVGASILIMP KFEIGTLLGL IEKYKVSIAP VVPPVMLAIA R--SPDLDKH DLSSLRMIKS GGAPLGKELE DTVRAKFP-Q ARLGQGYGMT  333
Pt X Pd 4CL1 AAC39366 557 AA    252 RVGSAILLMQ KFEIVTLMEL VQKYKVTIAP FVPPVVLAVA K--CPVVDKY DLSSIRTVMS GAAPMGKELE DTVRAKLP-N AKLGQGYGMT  338
Nt 4CL1 O24145 547 AA           255 RVGAAILIMQ KFDIAPFLEL IQKYKVSIGP FVPPIVLAIA K--SPIVDSY DLSSVRTVMS GAAPLGKELE DAVRTKFP-N AKLGQGYGMT  341
St AAD40664 545 AA              253 RVGAAILIMQ KFDIAQFLEL IPKHKVTIGP FVPPIVLAIA K--SPLVHNY DLSSVRTVMS GAAPLGKELE DAVRAKFP-N AKLGQGYGMT  339
Pc 4CL1 P14912 544 AA           251 RAGVTILIMQ KFDIVPFLEL IQKYKVTIGP FVPPIVLAIA K--SPVVDKY DLSSVRTVMS GAAPLGKELE DAVRAKFP-N AKLGQGYGMT  337
Vp O24540 553 AA                259 RAGSGILIMQ KFEIVPFLEL IQKYKVTIGP FVPPIVLAIA K--STVVDNY DLSSVRTVMS GAAPLGKELE DAVRAKFP-N AKLGQGYGMT  345
At 4CL-LK At419010 NP_193636    274 SLGSTIVVMK RFDASDVVNV IERFKITHFP VVPPMLMALT KKAKGVCGEV -FKSLKQVSS GAAPLSRKFI EDFLQTLP-H VDLIQGYGMT  361
At At4g05160 NP_192425 544 AA   257 QRGNALVSMA RFELELVLKN IEKFRVTHLW VVPPVFLALS K--QSIVKKF DLSSLKYIGS GAAPLGKDLM EECGRNIP-N VLLMQGYGMT  343
At At5g63380 NP_201143 562 AA   271 SLGETLVLLG RFELEAMFKA VEKYKVTGMP VSPPLIVALV K--SELTKKY DLRSLRSLGC GGAPLGKDIA ERFKQKFP-D VDIVQGYGLT  357
At At5g38120 NP_198628 550 AA   264 ALGTTVVILP RFDLGEMMAA VEKYRATTLI LVPPVLVTMI NKADQIMKKY DVSFLRTVRC GGAPLSKEVT QGFMKKYP-T VDVYQGYALT  352
At At1g20480 NP_173472 565 AA   276 ALGWTIVVLP KFDMAKLLSA VETHRSSYLS LVPPIVVAMV NGANEINSKY DLSSLHTVVA GGAPLSREVT EKFVENYP-K VKILQGYGLT  364
At At1g20510 AAP3021 546 AA      255 AYGSTIIVLS KFEMHEMMSA IGKYQATSLP LVPPILVAMV NGADQIKAKY DLSSMHTVLC GGAPLSKEVT EGFAEKYP-T VKILQGYGLT  343

                                                                                  BOX II
                                                                             E401 ▬▬▬▬ C403
Pt X Pd AAC39366 557 AA         339 EAGPVLSMCL AFAKEPFEIK SGACGTVVRN AEMKIVDPD- TGRSLPRNQS GEICIRGSQI MKGYLNDPEA TERTVDNDGW LHTGDIGYID  427
Nt O24145 547 AA                 342 EAGPVLAMCL AFAKEPFDIK SGACGTVVRN AEMKIVDPD- TGCSLPRNQP GEICIRGDQI MKGYLNDPEA TTRTIDKEGW LHTGDIGFID  430
Af XP_753401 592 AA             365 EVGVVFQTRY GEQGN----- PGSIGRLLPG YEVRLVGQD- GNVVQGESRP GELYVRGSGV LMSYKGRNDA K----DAYGW FRTGDVAYVQ  444
An AfeA AAP13094 583 AA         358 EVGVVFQNRY GEPGN----- AASIGRLQPG YEIRLVGAD- GNLVADDNKP GELYVRGPGL LTCYRGRDDA K----DSQGW FRTGDVAYVN  437
Ao BAE62962 589 AA              363 EVGVVFQNRY GQPGN----- SGSIGRLVPG YEIRLVGQD- GNLVLDDNKP GELFVRGNGL LTGYKGRDDA K----DAQGW FRTGDVAYVN  442
At 4CL-LK At3g21230 AAP03020    366 ESG-TVAKSL AFAKNPFKTK SGACGTVIRN AEMKVVDTE- TGISLPRNKS GEICVRGHQL MKGYLNDPEA TARTIDKDGW LHTGDIGFVD  453
At 4CL4 Q9LU36 570 AA           366 ESG-TVAKSL AFAKNPFKTK SGACGTVIRN AEMKVVDTE- TGISLPRNKS GEICVRGHQL MKGYLNDPEA TARTIDKDGW LHTGDIGFVD  453
At 4CL3 Q9S777 561 AA           361 EAGPVLSMSL GFAKEPIPTK SGSCGTVVRN AELKVVHLE- TRLSLGYNQP GEICIRGQQI MKEYLNDPEA TSATIDEEGW LHTGDIGYVD  449
Le BAA08366 585 AA              379 EAGPVLSMSP SFAKHPYPAK SGSCGTVVRN ADLKVIDPE- TGSSLGRNQP GEICIRGEQI MKGYLNDPEA TARTVDIEGW LHTGDIGYVD  467
Sa NP_825041 524 AA              323 ELSPGTHVVP LNAVNP---P PGTVGKLVAG TEMRILSLDD PDQDLPVGEA GEIAIRGPQV MKGYLGRPEA TAAMIDEDGW LHTGDVGRVD  409
Gm 4CL3 AAC97500 570 AA          363 EAGPVLSMCL GFAKQPFPTK SGSCGTVVRN AELRVVDPE- TGRSLGYNQP GEICIRGQQI MKGYLNDEKA TALTIDSEGW LHTGDVGYVD  451
Pt AAC24504 570 AA              364 EAGPVLSMCL AFSKQPFPTK SGSCGTVVRN AELKVIDPE- TGRSLGYNQP GEICIRGSQI MKGYLNDAEA TANTIDVEGW LHTGDIGYVD  452
At 4CL1 Q42524 561 AA           358 EAGPVLAMSL GFAKEPFPVK SGACGTVVRN AEMKIVDPD- TGDSLSRNQP GEICIRGHQI MKGYLNNPAA TAETIDKDGW LHTGDIGLID  446
At 4CL2 Q9S725 556 AA           351 EAGPVLAMSL GFAKEPFPVK SGACGTVVRN AEMKILDPD- TGDSLPRNKP GEICIRGNQI MKGYLNDPLA TASTIDKDGW LHTGDVGFID  439
Pto AAL56850 536 AA             334 EAGPVLAMCL AFAKEPFDIK PGACGTVVRN AEMKIVDPE- TGASLPRNQP GEICIRGDQI MKGYLNDPEA TSRTIDKEGW LHTGDIGYID  422
Pt X Pd 4CL1 AAC39366 557 AA    339 EAGPVLSMCL AFAKEPFEIK SGACGTVVRN AEMKIVDPD- TGRSLPRNQS GEICIRGSQI MKGYLNDPEA TERTVDNDGW LHTGDIGYID  427
Nt 4CL1 O24145 547 AA           342 EAGPVLAMCL AFAKEPFDIK SGACGTVVRN AEMKIVDPD- TGCSLPRNQP GEICIRGDQI MKGYLNDPEA TTRTIDKEGW LHTGDIGFID  430
St AAD40664 545 AA              340 EAGTVLTMCL AFAKEPFDIK SGACGTVVRN AEMKIVDPD- TGCSLPRNQP GEICIRGDQI MKGYLNDPEA TARTIEEEGW LHTGDIGFID  428
Pc 4CL1 P14912 544 AA           338 EAGPVLAMCL AFAKEPYEIK SGACGTVVRN AEMKIVDPE- TNASLPRNQR GEICIRGDQI MKGYLNDPES TRTTIDEEGW LHTGDIGFID  426
Vp O24540 553 AA                346 EAGPVLAMCL AFAKEPFDIK SGACGTVVRN AEMKIVDPE- TGSSLPRNHP GEICIRGDQI MKGYLNDPEA TARTIDKEGW LHTGDIGYID  434
At 4CL-LK At419010 NP_193636    362 ESTAVGTRGF NSEKL---SR YSSVGLLAPN MQAKVVDWS- SGSFLPPGNR GELWIQGPGV MKGYLNNPKA TQMSIVEDSW LRTGDIAYFD  447
At At4g05160 NP_192425 544 AA   344 ETCGIVSVED PRLGK---RN SGSAGMLAPG VEAQIVSVE- TGKSQPPNQQ GEIWVRGPNM MKGYLNNPQA TKETIDKKSW VHTGDLGYFN  429
At At5g63380 NP_201143 562 AA   358 ESSGPAASTF GPEEM---VK YGSVGRISEN MEAKIVDPS- TGESLPPGKT GELWLRGPVI MKGYVGNEKA SAETVDKEGW LKTGDLCYFD  443
At At5g38120 NP_198628 550 AA   353 ESNGAGASIE SVEES---RR YGAVGLLSCG VEARIVDPN- TGQVMGLNQT GELWLKGPSI AKGYFRNEEE ---IITSEGW LKTGDLCYID  435
At At1g20480 NP_173472 565 AA   365 ESTAIAASMF NKEET---KR YGASGLLAPN VEGKIVDPD- TGRVLGVNQT GELWIRSPTV MKGYFKNKEA TASTIDSEGW LKTGDLCYID  450
At At1g20510 AAP3021 546 AA      344 ESTGIGASTD TVEES---RR YGTAGKLSAS MEGRIVDPV- TGQILGPKQT GELWLKGPSI MKGYFSNEEA TSSTLDSEGW LRTGDLCYID  429

                                     BOX L
                                R449 ▬▬ K455 K457
Pt X Pd AAC39366 557 AA         428 GDDELFIVDR LKELIKYKGF QVAPAELEAM LIAHPDISDC AVVPMKDEA- AGEVPIAFVV RAN---GSKI TEDEIKQYIS KQVVFYKRIS  513
Nt O24145 547 AA                 431 EDDELFIVDR LKELIKYKGF QVAPAEIEAL LLNHPNISDA AVVPMKDEQ- AGEVPVAFVV RSN---GSAI TEDEVKDFIS KQVIFYKRVK  516
Af XP_753401 592 AA             444 -NGQYFIVGR TKELIKVRGW QVAPAELEAV LLKHPGIEDA AVTGVTSKDG STELPRAFVV RAKGPAANRL TAEEVYQFAR RQLASYKALD  533
An AfeA AAP13094 583 AA         437 -DGLYYIVGR TKELIKVRGW QVAPAELESI LLKHPGIEDA AVTGVTSKDG STEVPRAFVV RSKTLSGARL TSQEVYLFCR RQLASYKALD  526
Ao BAE62962 589 AA              442 -NGLYYIVGR TKELIKVRGW QVAPAELEAV LLKHPGIQDA AVTGVTSKDG STEVPRAFVV RTRQPAGMQL TSEEVYMYCR RQLASYKALD  531
At 4CL-LK At3g21230 AAP03020    454 DDDEIFIVDR LKELIKFKGY QVAPAELEAL LISHPSIDDA AVVAMKDEV- ADEVPVAFVA RSQ---GSQL TEDDVKSYVN KQVVHYKRIK  539
At 4CL4 Q9LU36 570 AA           454 DDDEIFIVDR LKELIKFKGY QVAPAELEAL LISHPSIDDA AVVAMKDEV- ADEVPVAFVA RSQ---GSQL TEDDVKSYVN KQVVHYKRIK  539
At 4CL3 Q9S777 561 AA           450 EDDEIFIVDR LKEVIKFKGF QVPPAELESL LINHHSIADA AVVPQNDEV- AGEVPVAFVV RSN---GNDI TEEDVKEYVA KQVVFYKRLH  535
Le BAA08366 585 AA              468 DDDEVFIVDR VKELIKFKGF QVPPAELEAL LISHPNIADA AVVPQKDAA- AGEVPVAFVV PSND--GFEL TEEAVKEFIS KQVVFYKRLH  554
Sa NP_825041 524 AA              410 ADGWLFVVDR VKELIKYKGF QVAPAELEAL LLTHPKIADA AVIGVYNDD- NNEVPHAHVV RQPS--AADL SAGEVMMYVA ERVAPYKRIR  496
Gm 4CL3 AAC97500 570 AA          452 EDDEIFIVDR VKELIKYKGF QVPPAELEGL LVSHPSIADA AVVPQKDVA- AGEVPVAFVV RSN---GFDL TEEAVKEFIA KQVVFYKRLH  537
Pt AAC24504 570 AA              453 DDDEIFIVDR VKEIIKFKGF QVPPAELEAL LVNHPSIADA AVVPQKDEV- AGEVPVAFVV RSD---DLDL SEEAVKEYIA KQVVFYKKLH  538
At 4CL1 Q42524 561 AA           447 DDDELFIVDR LKELIKYKGF QVAPAELEAL LIGHPDITDV AVVAMKEEA- AGEVPVAFVV KSK---DSEL SEDDVKQFVS KQVVFYKRIN  532
At 4CL2 Q9S725 556 AA           440 DDDELFIVDR LKELIKYKGF QVAPAELESL LIGHPEINDV AVVAMKEED- AGEVPVAFVV RSK---DSNI SEDEIKQFVS KQVVFYKRIN  525
Pto AAL56850 536 AA             423 DDDELFIVDR LKELIKYKGF QVAPAELEAL LIAHPEISDA AVVGMKDED- AGEVPVAFAV KSE---KSQA TEDEIKQYIS KQVIFYKRIK  508
Pt X Pd 4CL1 AAC39366 557 AA    428 GDDELFIVDR LKELIKYKGF QVAPAELEAM LIAHPDISDC AVVPMKDEA- AGEVPIAFVV RAN---GSKI TEDEIKQYIS KQVVFYKRIS  513
Nt 4CL1 O24145 547 AA           431 EDDELFIVDR LKELIKYKGF QVAPAEIEAL LLNHPNISDA AVVPMKDEQ- AGEVPVAFVV RSN---GSAI TEDEVKDFIS KQVIFYKRVK  516
St AAD40664 545 AA              429 DDDELFIVDR LKELIKYKGF QVAPAELEAL LINHPDISDA AVVPMIDEQ- AGEVPVAFVV RSN---GSTI TEDEVKDFIS KQVIFYKRIK  514
Pc 4CL1 P14912 544 AA           427 DDDELFIVDR LKEIIKYKGF QVAPAELEAL LLTHPTISDA AVVPMIDEK- AGEVPVAFVV RTN---GFTT TEEEIKQFVS KQVVFYKRIF  512
Vp O24540 553 AA                435 DDDELFIVDR LKELIKYKGF QVAPAELEAL LLTHPCISDA AVVPMKDEA- AGEVPVAFVV KSN---GHNI TEDEIKQFIS KQVIFYKRIN  520
At 4CL-LK At419010 NP_193636    448 EDGYLFIVDR IKEIIKYKGF QIAPADLEAV LVSHPLIIDA AVTAAPNEE- CGEIPVAFVV RRQ---ETTL SEEDVISYVA SQVAPYRKVR  533
At At4g05160 NP_192425 544 AA   430 EDGNLYVVDR IKELIKYKGF QVAPAELEGL LVSHPDILDA VVIPFPDEE- AGEVPIAFVV RSP---NSSI TEQDIQKFIA KQVAPYKRLR  515
At At5g63380 NP_201143 562 AA   444 SEDFLYIVDR LKELIKYKAY QVPPVELEQI LHSNPDVIDA AVVPFPDED- AGEIPMAFIV RKP---GSNL NEAQIIDFVA KQVTPYKKVR  529
At At5g38120 NP_198628 550 AA   436 NDGFLFIVDR LKELIKYKGY QVPPAELEAL LLNHPDILDA AVIPFPDKE- AGQFPMAYVA RKP---ESNL CEKKVIDFIS KQVAPYKKIR  521
At At1g20480 NP_173472 565 AA   451 GDGFVFVVDR LKELIKCNGY QVAPAELEAL LLAHPEIADA AVIPIPDMK- AGQYPMAYIV RKV---GSNL SESEIMGFVA KQVSPYKKIR  536
At At1g20510 AAP3021 546 AA      430 EDGFIFVVDR LKELIKYKGY QVAPAELEAL LLTHPEITDA AVIPFPDKE- VGQFPMAYVV RKT---GSSL SEKTIMEFVA KQVAPYKRIR  515
```

```
                                          K540
Pt X Pd AAC39366 557 AA          513 -RVFFTEAIP KAPSGKILRK DLRARLATGD FLIKFQHDTY MQKQQ----- --------- 557
Nt O24145 547 AA                 516 -RVFFVETVP KSPSGKILRK DLRARLAAG- VPN------- ---------- --------- 547
Af XP_753401 592 AA              534 GGIVFVEEIP RTASGKIQRF KLSQMNTYRD MVSSLLARFK GAASGLGGLG LVHKGRVAV 592
An AfeA AAP13094 583 AA          527 GGIIFVEEIP RTASGKIQRF KLTQMNTYRE IVSSLLARFR GAS--LQSVG IMHGGRIAV 583
Ao BAE62962 589 AA               532 GGVIFVEEIP RTASGKIQRF KLTQMNTYRE IVSSLLARFK GTSG-LQTVG LLHQGRITV 589
At 4CL-LK At3g21230 AAP03020     539 -MVFFIEVIP KAVSGKILRK DLRAKLETMC SK-------- ---------- --------- 570
At 4CL4 Q9LU36 570 AA            539 -MVFFIEVIP KAVSGKILRK DLRAKLETMC SK-------- ---------- --------- 570
At 4CL3 Q9S777 561 AA            535 -KVFFVASIP KSPSGKILRK DLKAKLC--- ---------- ---------- --------- 561
Le BAA08366 585 AA               554 -KVYFVHSIP KSPSGKILRK DLRAKLAAAA SS-------- ---------- --------- 585
Sa NP_825041 524 AA               496 -HVTFLDEVP RAASGKILRR QLRDLREHS- ---------- ---------- --------- 524
Gm 4CL3 AAC97500 570 AA          537 -KVYFVHAIP KSPSGKILRK DLRAKLETAA TQTP------ ---------- --------- 570
Pt AAC24504 570 AA               538 -KVFFVHSIP KSASGKILRK DLRAKLATAT TMS------- ---------- --------- 570
At 4CL1 Q42524 561 AA            532 -KVFFTESIP KAPSGKILRK DLRAKLANGL ---------- ---------- --------- 561
At 4CL2 Q9S725 556 AA            525 -KVFFTDSIP KAPSGKILRK DLRARLANGL MN-------- ---------- --------- 556
Pto AAL56850 536 AA              508 -RVFFIEAIP KAPSGKILRK NLKEKLPGI- ---------- ---------- --------- 536
Pt X Pd 4CL1 AAC39366 557 AA     513 -RVFFTEAIP KAPSGKILRK DLRARLATGD FLIKFQHDTY MQKQQ----- --------- 557
Nt 4CL1 O24145 547 AA            516 -RVFFVETVP KSPSGKILRK DLRARLAAG- VPN------- ---------- --------- 547
St AAD40664 545 AA               514 -RVFFVETVP KSPSGKILRK DLRARLAAG- ISN------- ---------- --------- 545
Pc 4CL1 P14912 544 AA            512 -RVFFVDAIP KSPSGKILRK DLRARIASGD LPK------- ---------- --------- 544
Vp O24540 553 AA                 520 -RVFFVEAIP KAPSGKILRK DLRARLAAAA LPTN------ ---------- --------- 553
At 4CL-LK At419010 NP_193636     533 -KVVMVNSIP KSPTGKILRK ELKRILTNSV SSRL------ ---------- --------- 566
At At4g05160 NP_192425 544 AA    515 -RVSFISLVP KSAAGKILRR ELVQQVRS-- --KM------ ---------- --------- 544
At At5g63380 NP_201143 562 AA    529 -RVAFINAIP KNPAGKILRR ELTKIAVDGN ASKL------ ---------- --------- 562
At At5g38120 NP_198628 550 AA    521 -KVAFIDSIP KTPSGKTLRK DLIKFAIS-- --KI------ ---------- --------- 550
At At1g20480 NP_173472 565 AA    536 -KVTFLASIP KNPSGKILRR ELTKLTTS-- --KL------ ---------- --------- 565
At At1g20510 AAP3021 546 AA      515 -KVAFVSSIP KNPSGKILRK DLIKIATSN- -SKL------ ---------- --------- 546
```

Figure S3. AfeA is a member of the superfamily of adenylate-forming enzymes. Proteins from plants, bacteria and fungi, showing the highest similarity to AfeA were aligned using ClustalW (Blosum 62). Important amino acids (see text) are indicated in orange or delimited by squares. Conserved domains are indicated with bars and the AMP-binding domain is between orange brackets. Substrate binding domains sbd I and sbd II are in blue and brown brackets, respectively. Clear C-terminal peroxisomal localization signals are indicated in lilac. The organism names are: *At*, *Arabidopsis thaliana*; *Sa*, *Streptomyces avermitilis*; *Af*, *Aspergillus fumigatus*; *An*, *Aspergillus nidulans*; *Ao*, *Aspergillus oryzae; Pb* X *Pd*, *Populos balsamifera* and *Populos deltoides* hybrid*; Nt, Nicotiana tabacum*; *St*, *Solanum tuberosum*; *Pc*, *Petroselinum crispum*; *Vp*, *Vanilla planifolia*; *Pto*, *Populos tomentosa*; *Le*, *Lithospermum erythrorhizon; Gm*, *Glycine max*; *Pt*, *Populos tremuloides.*
