# Supplementary material for: The Adenylate-Forming Enzymes AfeA and TmpB Are Involved in Aspergillus nidulans Self-Communication during Asexual Development
Source: Front Microbiol. 2016 Mar 23;7:353. doi: 10.3389/fmicb.2016.00353 (PMC4804170; doi:10.3389/fmicb.2016.00353)
Supplement: Supplementary file 5 [file Image4.pdf]

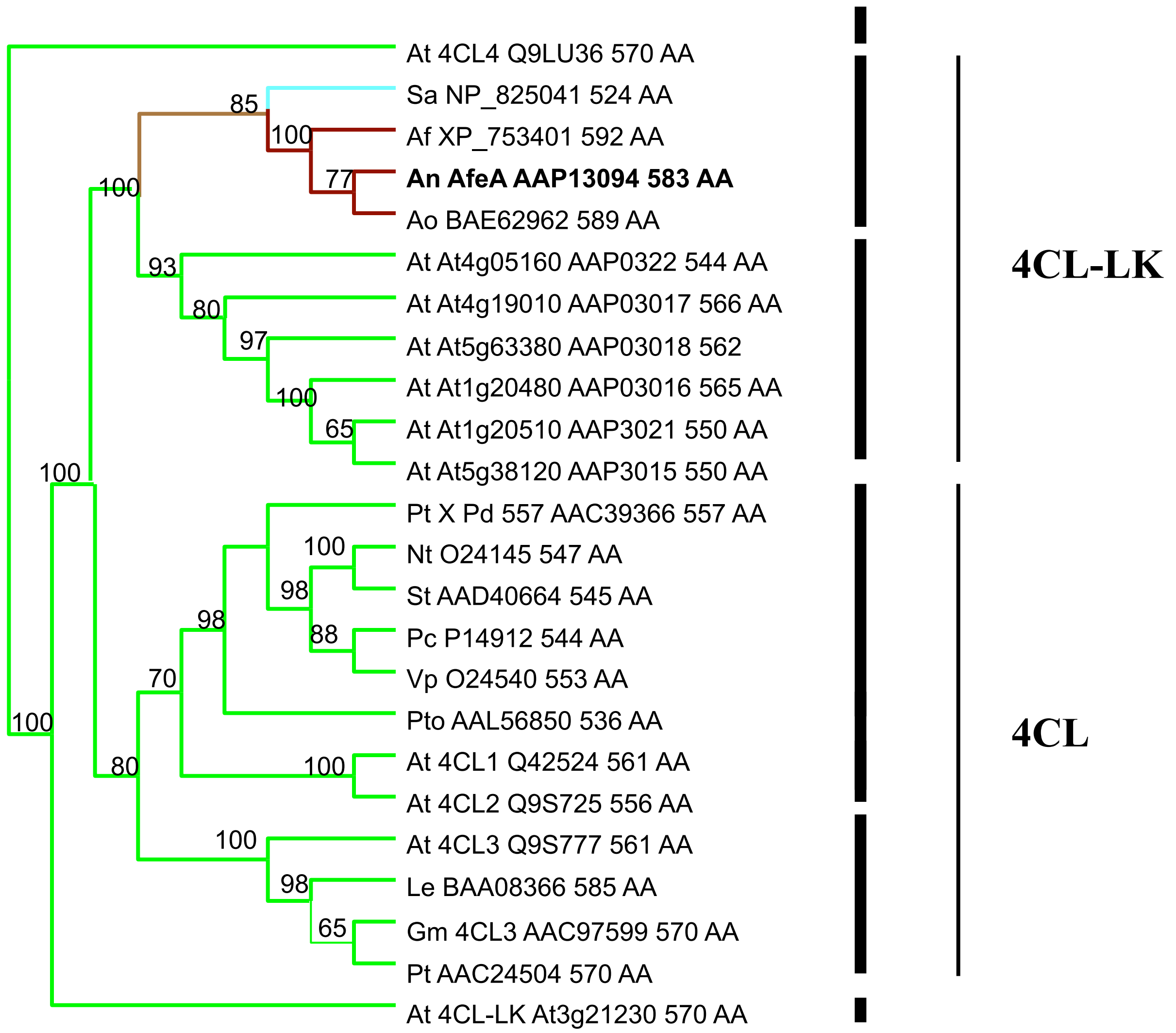

Figure S4. Phylogenetic analysis of proteins from plants, bacteria and fungi, showing the highest similarity to AfeA. AfeA homologs were aligned using ClustalW (blosum 30) and the program MacVector 7.2. The tree was generated with the Neighbor Joining method. Node numbers are percentage of 2000 bootstrap replicates (Poisson correction) in which that node was recovered. Protein GenBank access and number of amino acids are indicated. 4CL and 4CL-LK include plant coumarate ligase and coumarate ligase-like enzymes, respectively. AfeA fungal and bacterial homologs are indicated in brown and blue color, respectively. Organisms full names are: *At*, *Arabidopsis thaliana*; *Sa*, *Streptomyces avermitilis*; *Af*, *Aspergillus fumigatus*; *An*, *Aspergillus nidulans*; *Ao*, *Aspergillus oryzae*; *Pb* X *Ps*, hybrid between *Populos balsamifera* and *Populos deltoides*; *Nt*, *Nicotiana tabacum*; *St*, *Solanum tuberosum*; *Pc*, *Petroselinum crispum*; *Vp*, *Vanilla planifolia*; *Pto*, *Populos tomentosa*; *Le*, *Lithospermum erythrorhizon*; *Gm*, *Glycine max*; *Pt*, *Populos tremuloides*.
