# Supplementary material for: The Adenylate-Forming Enzymes AfeA and TmpB Are Involved in Aspergillus nidulans Self-Communication during Asexual Development
Source: Front Microbiol. 2016 Mar 23;7:353. doi: 10.3389/fmicb.2016.00353 (PMC4804170; doi:10.3389/fmicb.2016.00353)
Supplement: Supplementary file 6 [file Image5.pdf]

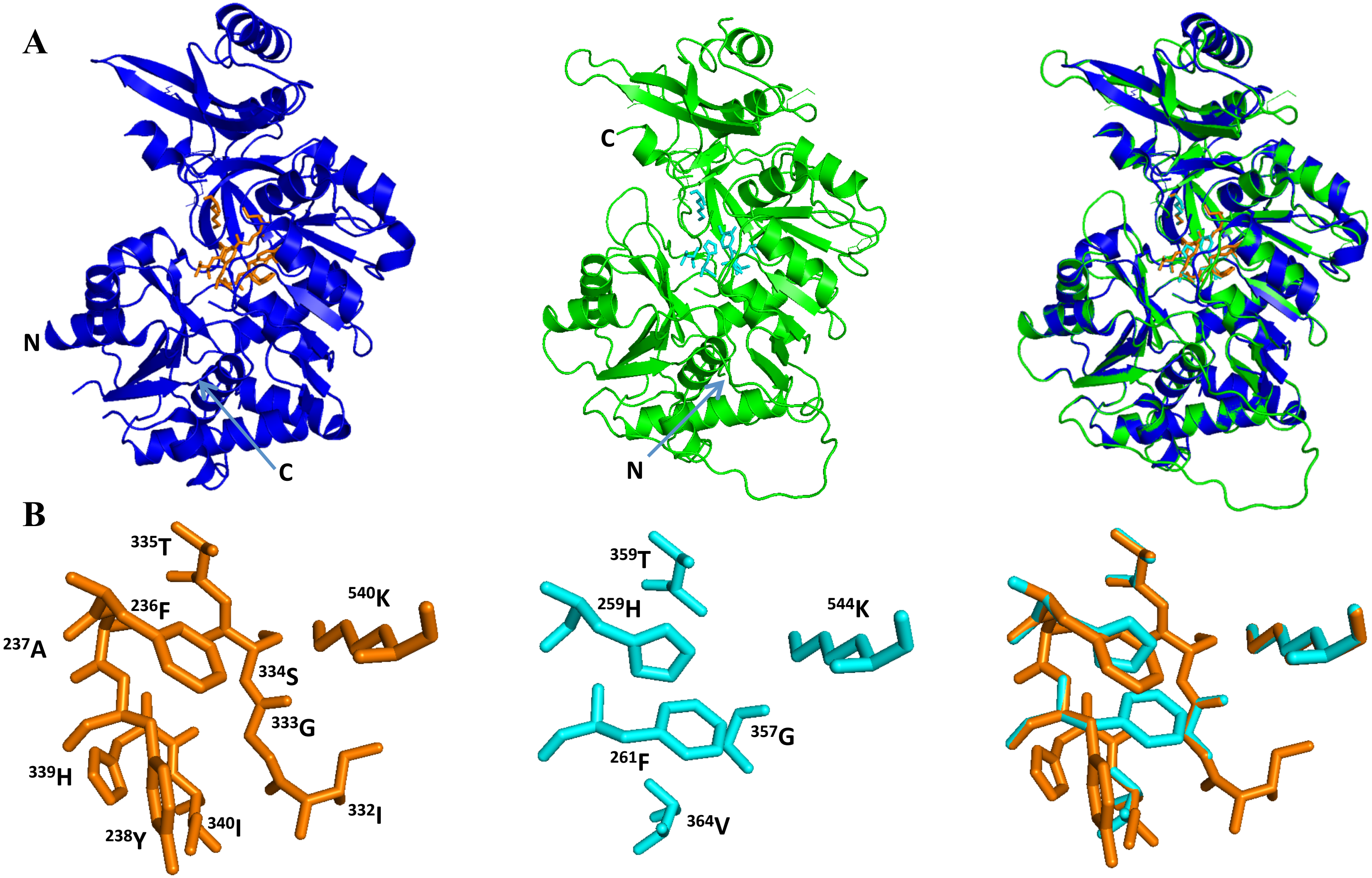

Figure S5. Modeling of AfeA using the crystal structure of Benzoate coA ligase (BCL) from *Burkholderia xenovorans* LB400. **(A)** Protein structures of BCL (left), AfeA model based on BCL (center) and BCL/AfeA superposition (right) **(B)** Expanded structural view (rotated about 90° to the right with respect to (A)) of key residues lining the substrate-binding pockets, in the same order as in (A). Structure modeling and model display were done using programs SWISS-MODEL and PyMOL Molecular Graphics System (1.7.4 version, Schrödinger, LLC), respectively. N and C indicate the protein amino and carboxy-terminal ends. BCL and AfeA substrate-binding pocket residues showing some overlap are indicated in B (central panel).
