# Supplementary material for: The Adenylate-Forming Enzymes AfeA and TmpB Are Involved in Aspergillus nidulans Self-Communication during Asexual Development
Source: Front Microbiol. 2016 Mar 23;7:353. doi: 10.3389/fmicb.2016.00353 (PMC4804170; doi:10.3389/fmicb.2016.00353)
Supplement: Supplementary file 7 [file Image6.pdf]

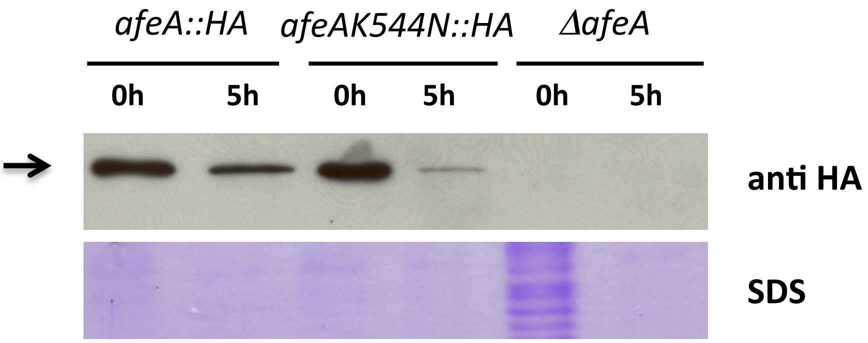

Figure S6. Expression of wild type and mutant AfeA tagged with HA. *S*trains TOS10 (*afeA::HA*), TOS16 (*afeAK544N::HA*) and CGS17 (*ΔafeA*) grown for 18 h in MM (0h of starvation) were transferred to MM without nitrogen for 5 hours. Mycelia harvested at indicated times was used to prepare total protein extracts for immunoblotting. Lower panel show the Coomasie-stained gel used for immunoblotting, as protein loading control. Arrow indicates the position of 60kDa pre-stained SDS–PAGE molecular weight marker (not sown).

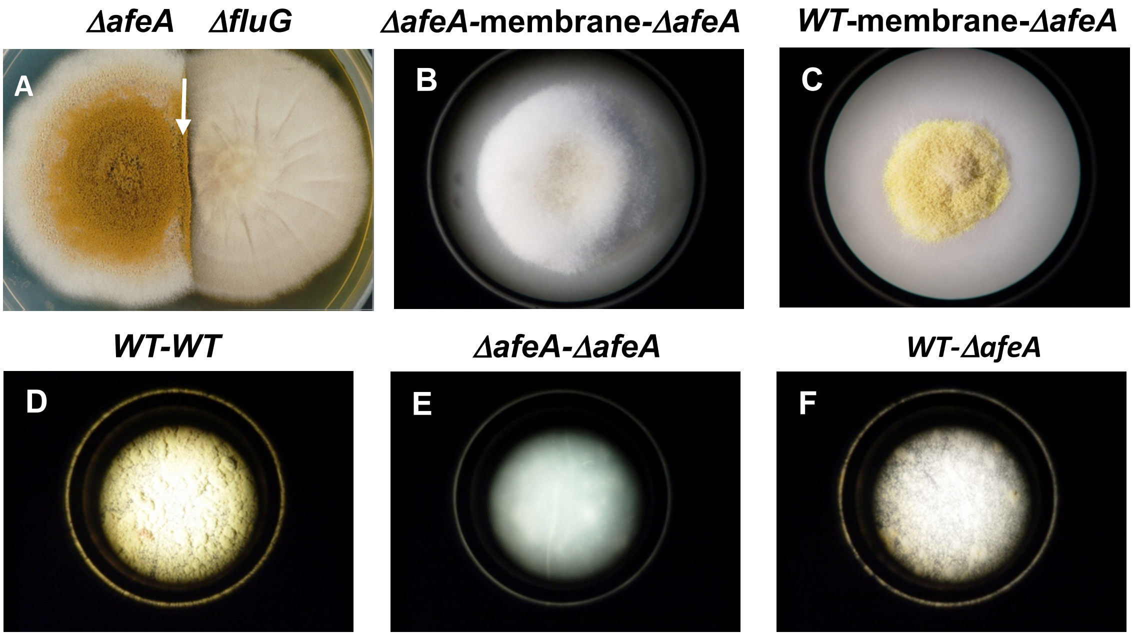

Figure S7. *ΔafeA* mutant conidiation defects are remediated by a contiguous *ΔfluG* mutant strain or by medium conditioned by a WT strain. **(A)** Yellow spore *ΔafeA* mutant strain TGS1 was inoculated next to green spore *ΔfluG* mutant strain RBN119 a line of wild type conidiophores was produced (white arrow) between both strains. **(B)** *ΔafeA* CGS17 mutant and **(C)** WT FGS26 green spore strains were grown for 18 h and then covered with a 0.4 µm Millipore membrane. TGS1 strain was point inoculated on top of the membrane from both strains and further grown for 72 h. **(D and F)** Wild type CLK43 or **(E)** TGS1 strains were grown for 24 h and then mycelium was removed and the medium was filtered using a 0.22 µm Millipore membrane. Filtered medium was inoculated with CLK43 **(D)** or TGS1 **(E-F)** conidia and grown for 45 h.
