# Supplementary material for: The Adenylate-Forming Enzymes AfeA and TmpB Are Involved in Aspergillus nidulans Self-Communication during Asexual Development
Source: Front Microbiol. 2016 Mar 23;7:353. doi: 10.3389/fmicb.2016.00353 (PMC4804170; doi:10.3389/fmicb.2016.00353)
Supplement: Supplementary file 8 [file Image7.pdf]

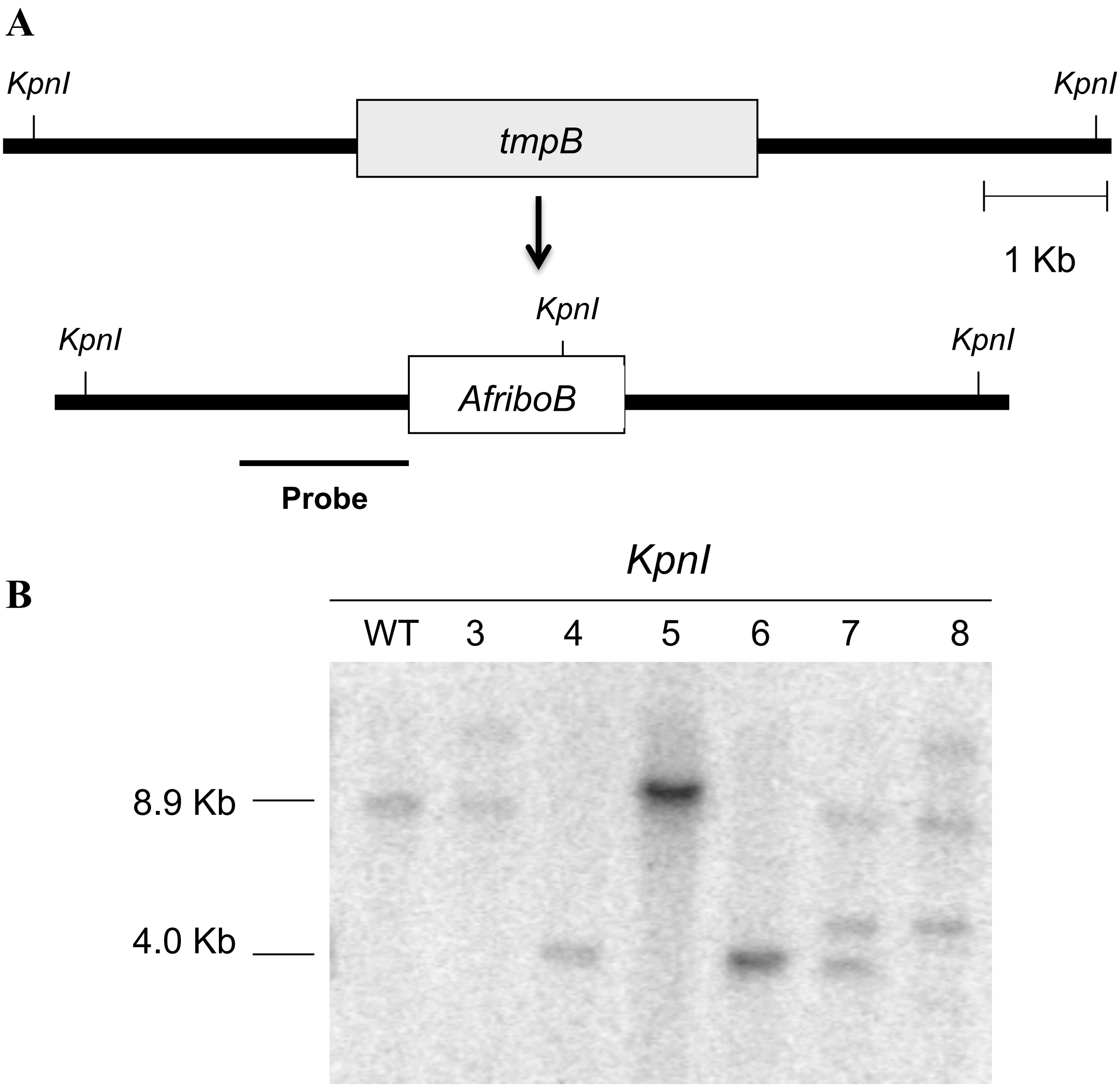


**Figure S8.** Deletion of the *tmpB* gene. **(A)** The *tmpB* ORF was replaced by the *AfriboB* gene, used as a selective marker. The deletion cassette was generated by double joint PCR, using primers 7dtmpAL and 8dtmpAL and used to transform strain A770. **(B)** Total DNA extracted from strains A770 (WT) and transformants 3-8 was digested with *KpnI* and used for Southern blot analysis, using the indicated *tmpB*-specific probe, amplified by PCR using primers 1tmpAL and 2tmpAL. The hybridization patterns of transformants 4 (TΔtmpB4) and 6 (strain TΔtmpB6) are consistent with the integration event illustrated in **(A)**.
