# Supplementary material for: The Adenylate-Forming Enzymes AfeA and TmpB Are Involved in Aspergillus nidulans Self-Communication during Asexual Development
Source: Front Microbiol. 2016 Mar 23;7:353. doi: 10.3389/fmicb.2016.00353 (PMC4804170; doi:10.3389/fmicb.2016.00353)
Supplement: Supplementary file 9 [file Image8.pdf]

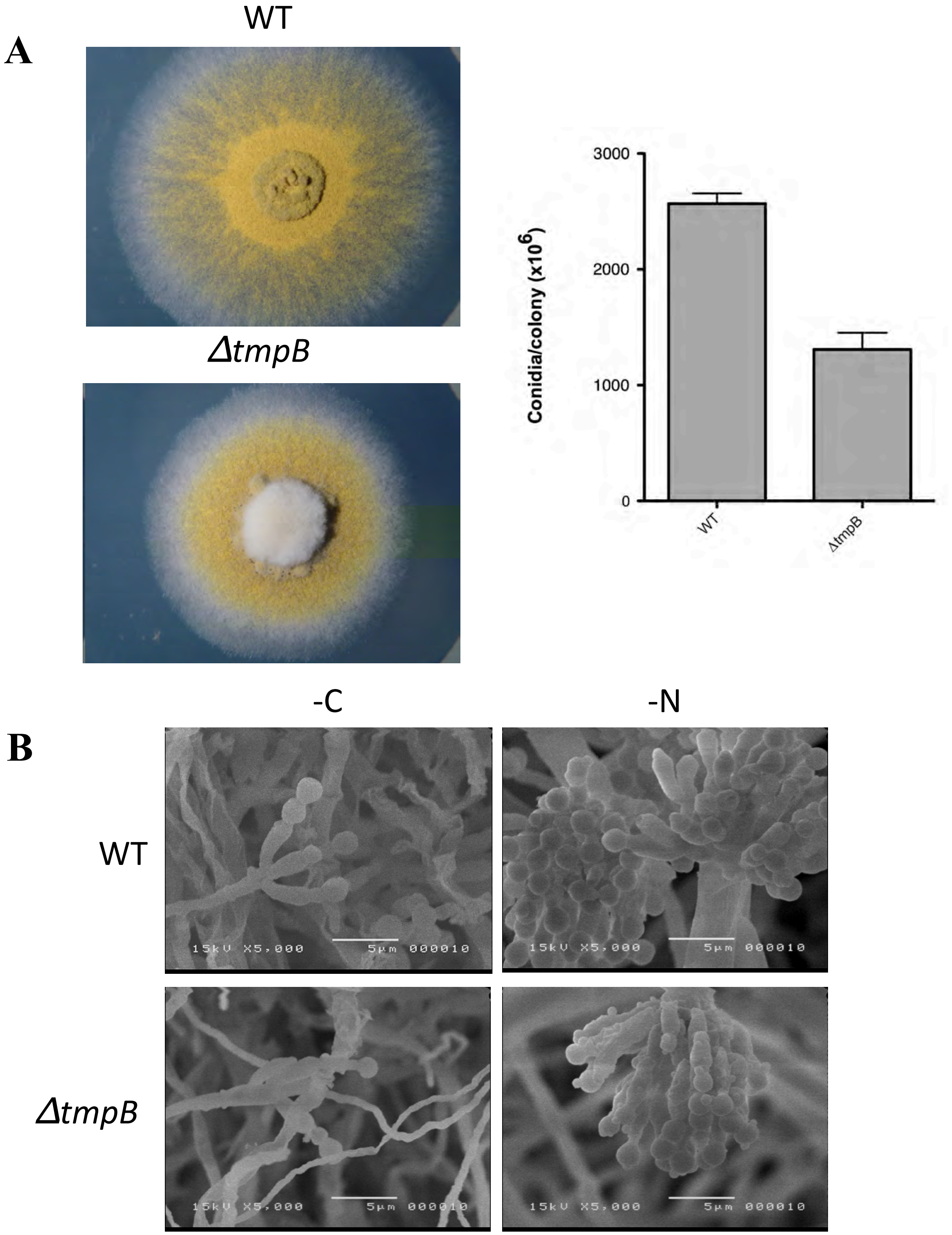

Figure S9. TmpB is required for normal air-induced conidiation but dispensable for nitrogen starvation-induced conidiation. **(A)** Strains CLK43 (WT) and CΔTMPBP1 (*ΔtmpB*) were point inoculated, grown for 72 h and photographed, and the total number of spores per colony was counted after five days. Bars represent standard deviation from three independent experiments. **(B)** The same strains as in **(A)** were grown in MM-glucose for 18 h and then shifted to MM lacking either glucose (-C) or nitrate (-N) for 20 h and then samples were processed for electron scanning microscopy.
