# Supplementary material for: The Adenylate-Forming Enzymes AfeA and TmpB Are Involved in Aspergillus nidulans Self-Communication during Asexual Development
Source: Front Microbiol. 2016 Mar 23;7:353. doi: 10.3389/fmicb.2016.00353 (PMC4804170; doi:10.3389/fmicb.2016.00353)
Supplement: Supplementary file 10 [file Image9.pdf]

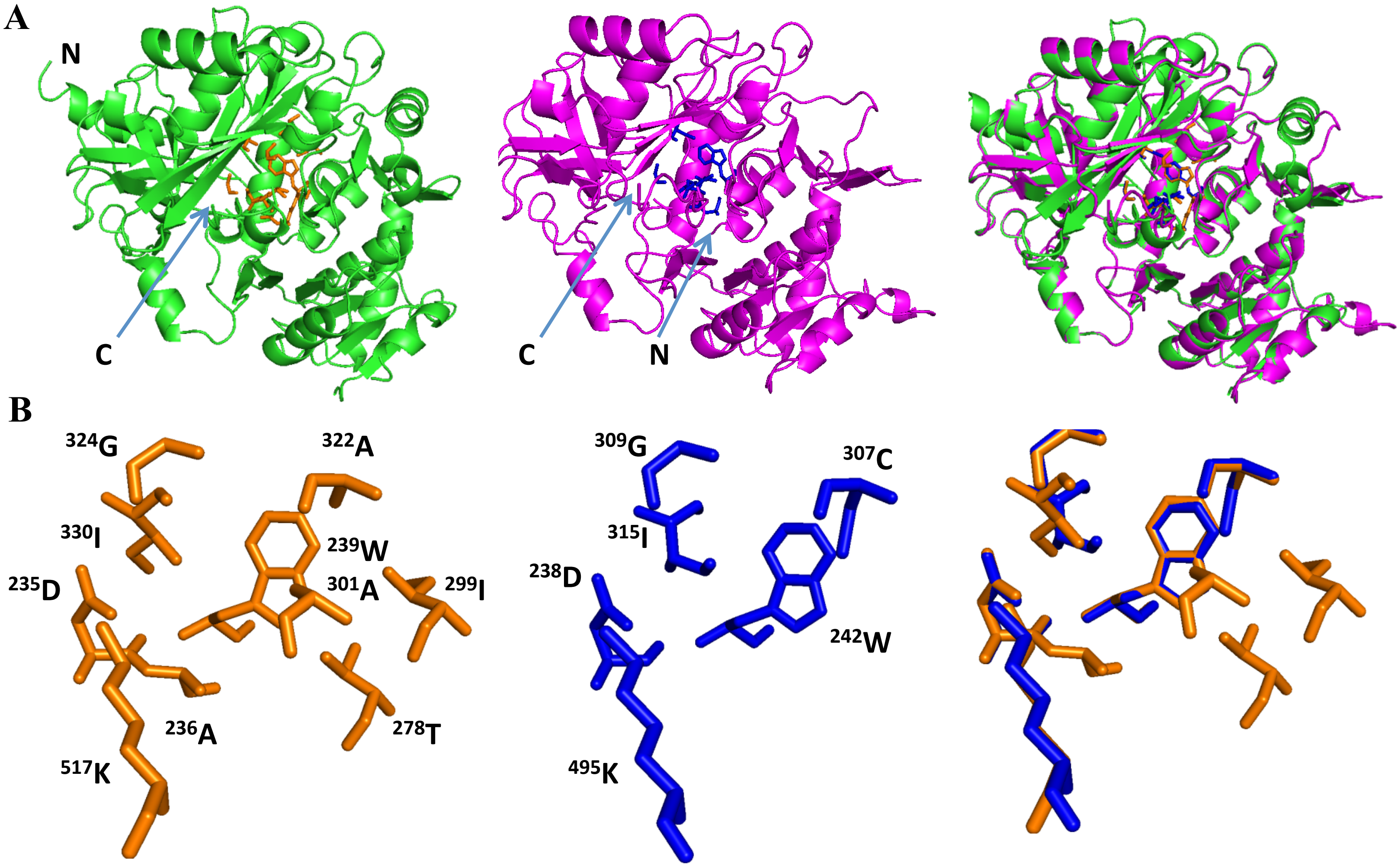

Figure S10. Modeling of TmpB adenylation domain using the crystal structure of the phenylalanine activating subunit of gramicidin synthetase 1 (PheA) from *Bacillus brevis*. **(A)** Protein structures of PheA (left), TmpB model based on PheA (center) and PheA/TmpB superposition (right). **(B)** Expanded rotated view of key residues lining the substrate-binding pockets, in the same order as in **(A)**. Structure modeling and model display were done using programs SWISS-MODEL and PyMOL Molecular Graphics System (1.7.4 version, Schrödinger, LLC), respectively. N and C indicate protein amino and carboxy-terminal ends. PheA and TmpB substrate-binding pocket residues that overlap well are indicated in B (central panel).
